# Impairment of oxidative metabolism compromises Rad51 recruitment and potentiates PARP inhibitor effectiveness in ovarian cancer

**DOI:** 10.1186/s13046-026-03641-6

**Published:** 2026-01-14

**Authors:** Laura Formenti, Francesca Abramo, Giulia Dellavedova, Valentina Dematteis, Alessandra Decio, Chiara Grasselli, Paola Fabbrizio, Laura Brunelli, Raffaella Giavazzi, Carmen Ghilardi

**Affiliations:** 1 Mario Negri Institute for Pharmacological Research IRCCS, Department of Experimental Oncology, Unit of Molecular Anti-Tumour Therapy, Milan, Italy; 2 Mario Negri Institute for Pharmacological Research IRCCS, Department of Experimental Oncology, Laboratory of Cancer Metastasis Therapeutics, Milan, Italy; 3 Mario Negri Institute for Pharmacological Research IRCCS, Department of Experimental Oncology, Unit of Immunopharmacology, Milan, Italy; 4 Mario Negri Institute for Pharmacological Research IRCCS, Department of Neuroscience, Laboratory of Neuromuscular Disorders, Milan, Italy; 5 Mario Negri Institute for Pharmacological Research IRCCS, Department of Environmental Health Sciences, Laboratory of Metabolites and Proteins in Translational Research, Milan, Italy; 6 Present Address: ITALFARMACO S.p.A., Cinisello Balsamo, Italy

**Keywords:** Mitochondria, PARP inhibitors, Ovarian cancer, DNA repair, OC-PDXs

## Abstract

**Background:**

The treatment of ovarian cancer has significantly improved since the introduction of PARP inhibitors (PARPi), small molecules designed to directly target and kill cancer cells with deficiencies in homologous recombination (HR) pathway. However, nearly half of patients present with HR-proficient tumors, rendering them not eligible for PARPi-based therapies and underscoring the urgent need for alternative treatment strategies.

**Methods:**

Oxidative metabolism has been altered either by silencing the mitochondria regulator PGC-1β or by using the OXPHOS inhibitor IACS-010759. The metabolic alterations were characterized by seahorse analysis and metabolomic profiling. DNA damage and repair were evaluated by immunofluorescence and confocal analysis. Efficacy and tolerability of the combination of PARP and OXPHOS inhibitors were investigated in preclinical trials employing patient-derived ovarian cancer xenografts.

**Results:**

Our findings reveal that PGC-1β silencing sensitizes ovarian cancer cells to PARPi by impairing oxidative metabolism, reducing succinate levels and decreasing Fen1 succinylation and SUMOylation. The impairment of these post-translational modifications hinders Fen1 activation and prevents the recruitment of Rad51, resulting in a HR-deficient-like phenotype. The translational relevance of the findings has been validated using the OXPHOS inhibitor IACS-010759, which synergizes with PARPi to inhibit cancer cell proliferation, while sparing normal cells. Furthermore, the combination therapy delays tumor progression in ovarian cancer xenografts not responsive to PARPi, independently from their HR status.

**Conclusions:**

Targeting mitochondrial metabolism depicts a novel mechanism to modulate DNA repair and enhance PARPi sensitivity. This approach broadens the therapeutic applicability of PARP inhibitors beyond HR-deficient tumors and offers promising avenues to overcome resistance in ovarian cancer treatment.

**Supplementary Information:**

The online version contains supplementary material available at 10.1186/s13046-026-03641-6.

## Background

Ovarian cancer (OC) is one of the most common and lethal gynecological neoplasm [1]. OC treatment has been transformed by the introduction of the angiogenesis inhibitor bevacizumab and, more recently, poly (ADP-ribose) polymerase (PARP) inhibitors (PARPi) [2, 3]. The latter are designed to inhibit PARP proteins and induce cell death in homologous recombination deficient (HRD) cancer cells, whose prototypes are *BRCA*-mutated cells [4]. PARPi selectively induce synthetic lethality, causing the formation of DNA double-strand breaks (DSBs) that cannot be repaired in HRD cells, while sparing homologous recombination proficient (HRP) cells [4, 5]. Currently, various PARPi have been approved in the clinic for the treatment of HRD OC patients [2, 3], who have shown great benefit from this therapy [6]. However approximately 40–50% of patients are not eligible for PARPi as HRP, and limited therapeutic options are available for them.

The importance of mitochondria and oxidative metabolism in OC has recently been highlighted [7–9]. Mitochondrial DNA content is significantly higher in ovarian tumors than in normal ovarian tissue [10] and is associated with higher activity of respiratory chain complexes and citrate synthase [11]. Moreover, oxidative phosphorylation (OXPHOS) appears to play a key role in the maintenance of OC stem cells and in the insurgence of chemoresistance [12, 13]. We recently reported that high expression of peroxisome proliferator-activated receptor gamma coactivator 1-alpha and beta (PGC-1α and PGC-1β) activates an OXPHOS gene program, leading to increased mitochondrial abundance, enhanced tricarboxylic acid cycling (TCA) and elevated cellular respiration, distinguishing ovarian tumors mostly depending on mitochondrial metabolism as the primary energy source. As a consequence of their addiction, these tumors are highly sensitive to pharmacological inhibition of OXPHOS with an electron transport chain complex I blocker [14].

Increasing evidence indicates the existence of a reciprocal interplay between oxidative metabolism, PARP activity, and DNA repair [15–17], which can influence the response to PARPi. To date, the majority of efforts have been focused on understanding the effect of PARP activation or inhibition on mitochondrial functions and oxidative metabolism [15, 18]. Additionally, ovarian and breast cancers, which display defects in homologous recombination (HR), rely on oxidative metabolism [19, 20]. In accordance, we observed that the oxidative phenotype is associated with the presence of *BRCA* defects [14]. Indeed, in a panel of 29 OC patient-derived xenografts (OC-PDXs), 78% of HRD OC-PDXs displayed the oxidative phenotype compared with 7% of HRP OC-PDXs. However, little is known about the influence of oxidative metabolism on DNA repair efficiency and its consequent effect on sensitivity to PARP inhibitors.

Here, we demonstrated that impairment of mitochondrial metabolism interferes with the machinery for the repair of DNA DSBs, thus leading to their accumulation and sensitization of HRP ovarian cancer cells to PARP inhibitors. We showed that pharmacological targeting of mitochondria can be successfully exploited for therapeutic purposes in combination with PARPi. Preclinical studies confirmed that the OXPHOS inhibitor IACS-010759 sensitized OC-PDXs poorly responsive to olaparib, delaying tumor progression and increasing the lifespan of tumor-bearing mice, thus opening the way for new therapeutic options for HRP and HRD PARPi-refractory ovarian cancers.

## Methods

### Cell culture

ID8 *Trp53*^*−/−*^-Luc (HRP; referred to as ID8) and ID8 *Trp53*^*−/−*^ *Brca2*^*−/−*^-Luc (HRD; referred to as ID8 *Brca2*^*−/−*^) are derivatives of the mouse ovarian carcinoma cell lines kindly provided by Susanne Downson and Iain McNeish (The University Court of the University of Glasgow; Glasgow) [21]. They were routinely cultured in DMEM high glucose medium (Euroclone), supplemented with 4% fetal bovine serum (FBS; Microgem), 2 mM L-glutamine (Euroclone), Insulin, Transferrin and Selenium solution 1X (ITS; Gibco) and 1 µg/ml of Blasticidin S HCl (Gibco).

The Caov-3 human ovarian adenocarcinoma cell line was purchased from ATCC and routinely cultured in DMEM high glucose medium supplemented with 20% FBS and 4 mM L-glutamine. The SK-OV-3 human ovarian adenocarcinoma cell line was obtained from the Division of Cancer Treatment, Tumor Repository (National Cancer Institute) and routinely cultured in RPMI-1640 medium (Euroclone), supplemented with 10% FBS and 2 mM L-glutamine.

HUVEC were isolated from umbilical cord veins and cultured in M199 medium (Euroclone) supplemented with 10% FBS, 10% newborn calf serum (Gibco), 2 mM L-glutamine, HEPES (Euroclone), heparin (15 U/ml; Teva Pharmaceutical Industries), and Endothelial Cell Growth Supplement (ECGS) (25 µg/ml; Alfa Aesar) in gelatin-coated dishes. UASMC smooth muscle cells were purchased from Biowhittaker and were routinely cultured in complete SmGM-2 medium (Lonza). HESkin human fibroblasts were isolated in-house and cultured in DMEM high glucose medium supplemented with 20% FBS and 2 mM L-glutamine.

Stocks of cell lines were authenticated by short-tandem repeat profiling (AmpFlSTR Identifiler Plus PCR Amplification Kit; Applied Biosystems) whenever possible, stored frozen in liquid nitrogen, and used within 2 (for primary cells) or 4 (for OC cell lines) weeks after thawing. The cells were routinely tested for mycoplasma contamination by PCR.

### Generation of PGC-1β knock down cells

shCTR ID8 and shPGC-1β ID8 #38 and #47 clones were obtained transfecting 1 × 10^6^ ID8 cells with 3.2 µg of either the MISSION TRC2 pLKO.1-puro non-Mammalian shRNA Control Plasmid DNA (Sigma-Aldrich) or the MISSION TRC2 pLKO.1-puro mouse *Ppargc1b* shRNA Plasmid DNA (Sigma-Aldrich) using the Gene Pulser II electroporator (Bio-Rad). Transfected cells were selected 48 hours (h) later using 5 µg/ml puromycin (Thermo Scientific Chemicals). Individual clones were then isolated by mechanical detachment of single colonies and further expanded in their culture medium supplemented with 2 µg/ml puromycin for subsequent characterization. PGC-1β silencing was verified by RT-qPCR and western blotting.

### Transfection with K200E-Fen1

1 × 10^6^ shPGC-1β ID8 #47 cells were transfected with 4 µg pCMV6-K200E-Fen1 (Origene), encoding for a mutant form of Fen1 (point mutation c.598 A > G) using the Gene Pulser II electroporator (Bio-Rad). Transfected cells were selected 48 h later and further expanded in their culture medium supplemented with using 1 mg/ml G418 Sulfate (Gibco). Expression of activated Fen1 was verified by western blotting.

### Drug preparation

IACS-010759 [14] has been provided by M.D. Anderson Cancer Center Institute for Applied Cancer Science and PARP inhibitors olaparib, veliparib or talazoparib have been purchased by Twin Helix.

For the in vitro experiments, all drugs were dissolved in DMSO (vehicle, Sigma-Aldrich). For the in vivo preclinical trials, IACS-010759 was dissolved in 0.5% methylcellulose (Sigma-Aldrich), whereas olaparib was formulated in 10% v/v DMSO in 30% w/v Kleptose (HP-β-CD; Sigma-Aldrich) in sterile MilliQ water.

### Proliferation assay

ID8 and its derivatives: 1,500 cells were plated in 96-well plates in RPMI-1640 supplemented with 4% FBS and 2 mM L-glutamine. After 24 h, the cells were treated with either IACS-010759, olaparib or both at the indicated concentrations (see Figure Legends) for 72 h. For rescue experiments, cells were treated with olaparib in absence/presence of 10 µM cell permeable succinate (NV118, Oroboros Instruments GmbH).

Human OC cells: 3,000 (SK-OV-3) or 5,000 (Caov-3) cells were plated in 96-well plates in RPMI-1640 supplemented with 2.5% FBS and 2 mM L-glutamine. After 48 h, the cells were treated with either IACS-010759, olaparib or both at the indicated concentrations (see Figure Legends) for 96 h.

Normal human cells: 2,500 (HESkin) or 3,000 (UASMC) or 4,000 (HUVEC) cells were plated in 96-well plates in their culture media. After 24 h, cells were treated with either IACS-010759 or olaparib or combined treatments at the indicated concentrations (see Figure Legends) for 72 h.

At the end of the treatments, the cells were stained with crystal violet (Sigma-Aldrich). After dye elution with a 1:1 solution of ethanol and 0.05 M sodium citrate (Sigma-Aldrich), absorbance was measured at 595 nm using a TECAN Infinite 200 PRO M plex. The percentage of cell proliferation was calculated as indicated in the Figure Legends.

For combination studies, data were analyzed using the isobologram method, as described in [22]. Briefly, the method relies on the calculation of the combined concentrations of IACS-010759 and olaparib that cause 30%, 50%, or 70% growth inhibition. In this way, multiple combinations of drug concentrations that achieved each effect were found, normalized to the IC_30_, IC_50_, or IC_70_ value of single drugs, and plotted in the isobologram. Combinations are defined additive if falling along the diagonal line connecting the IC_30_, IC_50_, or IC_70_ of the single drugs, synergistic when laying below the line, and antagonistic above.

The combination index (CI), for each combination of drug concentrations producing an effect “X” (i.e., 30%, 50%, and 70% growth inhibition), was calculated as follows: $$\small \begin{aligned} \mathrm{CI}=\mathrm{Conc}_{\mathrm{IACS}-010759\left(\mathrm X\right)}/{\mathrm{IC}}_{\mathrm{xIACS}-010759}+\mathrm{Conc}_{\mathrm{Olaparib}\left(\mathrm X\right)}/{\mathrm{IC}}_{\mathrm{xOlaparib}} \end{aligned}$$, where IC_x_ are the concentrations of each drug that would produce the effect X in monotherapy. The CI values obtained from all experiments were pooled and the mean and variance were calculated. A confidence band was calculated around each mean using a t distribution at the 90% probability level. Additivity was claimed when the value CI = 1 was inside the confidence band, synergism when the CI with its confidence band was < 1, antagonism when the CI with its confidence band was > 1.    

### Colony formation assay

One thousand ID8 cells or derivatives were plated in 6-well plates in RPMI-1640 supplemented with 4% FBS and 2 mM L-glutamine. After 48 h, the cells were treated with either IACS-010759, olaparib, or both at the indicated concentrations (see Figure Legends) for 6 days.

Two thousand SK-OV-3 or Caov-3 cells were plated in 6-well plates in RPMI-1640 medium supplemented with 2.5% FBS and 2 mM L-glutamine. After 5 days, the cells were treated with either IACS-010759, olaparib or both at the indicated concentrations (see Figure Legends) for 8 days.

Colonies were stained with crystal violet at the end of the treatment. The area occupied by colonies was quantified using the Entry Level image system (Immagini & Computers, Milan, Italy).

### Seahorse analysis

Ten thousand ID8 cells or derivatives were plated in XFe24 Cell Culture Microplates in culture medium and incubated overnight at 37 °C in 5% CO_2_. When specified, the cells were treated overnight with either DMSO or 25/100 nM IACS-010759. The Cell Mito Stress Test (Agilent Technologies) was performed according to the manufacturer’s instructions using 1 µM oligomycin, 2 µM FCCP and 1 µM rotenone and antimycin A. The oxygen consumption rate (OCR) was evaluated over a time course, before and after drug injection. OCR data (pmol/min) were normalized to cell number. The nuclei of cells were stained for 15 minutes (min) using Hoechst 33258 (1:750; Invitrogen #H3569), then cells were fixed for 15 min with 4% paraformaldehyde (PFA) (Thermo Scientific Chemicals). Eight fields/well were acquired using the ZOE Fluorescent Cell Imager (Bio-Rad), and the number of nuclei (cells) in each field was automatically counted using the ImageJ software. The mean of the 8 field counts was used to normalize the OCR values.

### Immunofluorescence and confocal analysis

Four thousand five hundred ID8 cells or derivatives were seeded in µ-Slide 8 Well high (Ibidi) in RPMI-1640 supplemented with 4% FBS and 2 mM L-glutamine and treated with either DMSO, 25 nM IACS-010759, 2 or 4 µM olaparib or their combination for 48 h. For the washout experiments, the cells were treated with 4 µM olaparib for 48 h and then processed 24 h after drug removal.

After treatment, the cells were fixed for 15 min with 4% PFA in PBS, permeabilized for 5 min with 0.1% Triton X-100 in PBS, and incubated for 10 min with cold methanol. Unspecific signals were blocked for 30 min with 1% bovine serum albumin (BSA; Sigma-Aldrich) in PBS, and the cells were incubated overnight at 4 °C with mouse anti-γH2AX (phospho S139) histone (1:500; Abcam #ab26350) and rabbit anti-Rad51 (1:1000; Abcam #ab133534). Cells were then incubated for 1 h at room temperature with goat anti-mouse IgG Alexa Fluor 488^®^ (1:500; Invitrogen #A-11017) for γH2AX and goat anti-rabbit IgG Alexa Fluor 594^®^ (1:500; Invitrogen #A-11012) for Rad51. Finally, the nuclei were counterstained for 10 min with Hoechst 33258 (1:750; Invitrogen #H3569). Three fields/well were acquired in sequential scanning mode using an A1 Nikon confocal running NIS Elements at 40X magnification. The number of γH2AX and co-localizing Rad51 foci were manually counted in Hoechst-positive nuclei using ImageJ software.

### Flow cytometry analysis

ID8 cells or derivatives were plated in RPMI-1640 supplemented with 4% FBS and 2 mM L-glutamine. After 24 h, the cells were treated as indicated in the Figure Legends with either DMSO, 25 nM IACS-010759, 2 or 4 µM olaparib or their combination for 48 h. At the end of the treatment, cells were harvested and fixed for 30 min with 1% methanol-free PFA (Electron Microscopy Sciences) in PBS and then overnight at 4 °C with 96% ethanol. Cells were then permeabilized for 10 min with 0.2% Triton X-100 in PBS-1%BSA and non-specific signals were blocked with 10% normal goat serum (Sigma-Aldrich) and 0.3 M glycine (Sigma-Aldrich) in PBS for 30 min. Cells were incubated for 45 min with mouse anti-γH2AX histone (1:100; Abcam #ab26350) and rabbit anti-Rad51 (1:1000; Abcam #ab133534) and then for 30 min with goat anti-mouse IgG Alexa Fluor 488^®^ (1:500; Invitrogen #A-11017) for γH2AX and goat anti-rabbit IgG Alexa Fluor 594^®^ (1:500; Invitrogen #A-11012) for Rad51. Finally, the DNA was stained for 30 min with a mixed solution of TO-PRO-3 (0.75 µM; Invitrogen #T3605) and 1% RNAse (Calbiochem) in PBS. The samples were analyzed using the CytoFLEX LX Flow Cytometer (Beckman Coulter). Offline analysis was performed using the Kaluza 1.2 software (Beckman Coulter). The fluorescence intensity was measured using median fluorescence intensity values.

### Metabolomics

ID8 cells and their derivatives were seeded and cultured in RPMI-1640 supplemented with 4% FBS and 2 mM L-glutamine for 72 h. When specified, 24 h after seeding, the cells were treated with either DMSO or 25 nM IACS-010759 for 48 h. Finally, the cells were rapidly rinsed in saline solution, quenched with liquid nitrogen, and stored at − 80 °C until analysis. Metabolites were extracted and analyzed by liquid chromatography high-resolution-mass spectrometry as previously described [23].

### RT-qPCR

Total RNA was isolated using the miRNeasy Mini Kit (Qiagen). RNA integrity was checked using the Agilent 6000 Nano Assay (Agilent Technologies) and RNA was reverse transcribed using the High-Capacity cDNA Reverse Transcription Kit (Applied Biosystem). qPCR was performed using TaqMan Universal PCR Master Mix (Applied Biosystems) and specific TaqMan Gene Expression Assays (*Actb*: Mm.PT.58.33540333; *Ppargc1b*: Mm.PT.58.8224107; IDT). *Ppargc1b* expression was normalized to *Actb* and calculated as relative expression (2^(−DDCt)^), using shCTR ID8 as a reference. The samples were run in triplicates.

### Immunoprecipitation

Total proteins were isolated using PIERCE IP lysis buffer (Thermo Scientific) supplemented with protease inhibitors (Sigma-Aldrich), cysteine (SUMO) protease inhibitor NEM and desuccinylating activity inhibitors (Suramin and MC3482, TargetMol) and quantified using the Bio-Rad protein assay kit (Bio-Rad). Fen1 was immunoprecipitated using the Dynabeads™ Protein A Immunoprecipitation Kit (Invitrogen) conjugated with the rabbit antibody anti-Fen1 (7.5 µg/500 µg of total protein; Bethyl laboratories #A300-255A) following manufacturer’s instructions. Fen1 post-translational modifications (succinylation and SUMOylation) were analyzed by western blotting.

### Western blot

Fifty µg total proteins or immunoprecipitated Fen1 were separated onto mPAGE™ 4–12% Bis-Tris Precast Gel (Millipore) and transferred to 0.2 μm PVDF membranes using standard protocols. Blots were blocked for 2 h at room temperature in either 5% non-fat dry milk or 5% BSA in TBS/0.1% Tween 20 (Sigma-Aldrich), and then incubated overnight at 4 °C with the appropriate primary antibody: anti-PGC-1β (1:5,000; Abcam #ab176328), anti-actin (1:20,000; Sigma-Aldrich #MAB1501), anti-succinyllysine (1:1,000; PTM BIO, #PTM-419), anti-SUMO-1 (1:1,000; Cytoskeleton, #ASM01), and anti-Fen1 (1:1,000; Invitrogen, #MA1-23228). The blots were then incubated for 1 h at room temperature with the appropriate secondary antibody: anti-rabbit IgG-HRP (1:12,000; Sigma-Aldrich #A6154) or anti-mouse IgG-HRP (1:10,000; Sigma-Aldrich #A4416). Signals were acquired using the Odyssey Fc Imaging System.

### Animals

Six- to eight-week-old female athymic Nude-*Foxn1*^*nu*^ mice were purchased from Envigo Laboratories and maintained under specific pathogen-free conditions, housed in isolated vented cages, and handled using aseptic procedures. All procedures involving animals and their care were conducted in conformity with the laws, regulations, and policies governing the care and use of laboratory animals: Italian Governing Law (D.lgs 26/2014, authorization number 19/2008-A issued March 6th 2008, by the Ministry of Health); Mario Negri Institutional regulations and policies that provide internal authorization for people conducting animal experiments (Quality Management System Certificate, UNI EN ISO 9001:2015 – regulation no. 6121); the NIH Guide for the Care and Use of Laboratory Animals (2011 edition); EU directives and guidelines (EEC Council Directive 2010/63/UE), and were in line with the guidelines for the welfare and use of animals in cancer research [24]. Animal studies were approved by the Mario Negri Institute Animal Care and Use Committee (IACUC) and authorized by the Italian Ministry of Health (authorization no. 393/2022- PR).

### Orthotopic ovarian cancer xenograft models

HOC76, HOC79, and HOC520, from a previously established and described panel of OC-PDXs [25], were stored as cryo-preserved stocks and propagated in mice by collecting malignant ascites from donor mice and intraperitoneal (i.p.) transplantation into the lower right quadrant of the mouse abdomen as a tumor suspension of 10 × 10^6^ cells. Tumor growth was evaluated based on abdominal distension and palpable tumor masses in the peritoneal cavity. Based on previous experiments, OC-PDX bearing mice were randomized (simple randomization) on the day estimated to correspond to 20–30% of their life expectancy (21-, 5-, or 10-days post-transplant for HOC76, HOC79, and HOC520, respectively), and groups were arbitrarily assigned to the different treatments as indicated in the Figure Legends.

IACS-010759 was administered orally at a dose of 2.5 mg/kg once daily. Olaparib was administered orally at a dose of 100 mg/kg once daily. All treatments were administered in a maintenance regimen, in cycles of 5 “on drug” days followed by 2 “off drug” days. Mice were monitored daily and humanely euthanized as soon as signs of distress/discomfort due to disease progression became apparent (visible abdominal swelling, hemorrhagic ascites, palpable abdominal tumor masses). The day of sacrifice was recorded as the survival time (ST) to generate Kaplan-Meier curves. For each group, the median ST (MST) was determined, and the increment in lifespan (ILS) was calculated as $$\tiny \scriptsize \begin{aligned} \mathrm{ILS}\%=100\times\left[\left(\mathrm{MST}\mathrm{drug} \mathrm{treated}\;-\;\mathrm{MST}\mathrm{vehicle}\mathrm{treated}\right)/\mathrm{MST}\mathrm{vehicle}\mathrm{treated}\;\right] \end{aligned}$$. At sacrifice, the abdominal tumor burden was evaluated as the volume of cancer cells in the harvested malignant effusion and as metastatic dissemination in representative organs and anatomical sites (e.g., uterus/ovary, diaphragm, liver, pancreas/omentum, and lymph nodes). Metastatization was rated by two independent scientists using an arbitrary scoring system (0 = no organ infiltration; 1 = small tumor masses; 2 = evident tumor masses; 3 = extensive organ infiltration; 4 = complete organ substitution) and for each mouse the sum of the ratings for all evaluated organs/anatomical sites was calculated.                

### Statistical analysis

Statistical analyses were done using the statistical tool implemented in GraphPad Prism 9.0 software (GraphPad, La Jolla, CA.). Data were tested for normal distribution using either the D’Agostino and Pearson or the Shapiro–Wilk tests. Nonparametric tests were used to analyze data that were not normally distributed. Normally distributed data were analysed using parametric tests, as indicated in each Figure Legend. All data met the assumptions of the statistical tests used. Statistical significance was set at *P* < 0.05. The detailed significance is reported in each corresponding Figure Legend.

## Results

### Impairment of mitochondria activity decreases the repair of DNA damage by reducing Fen1 succinylation

**Fig. 1:**
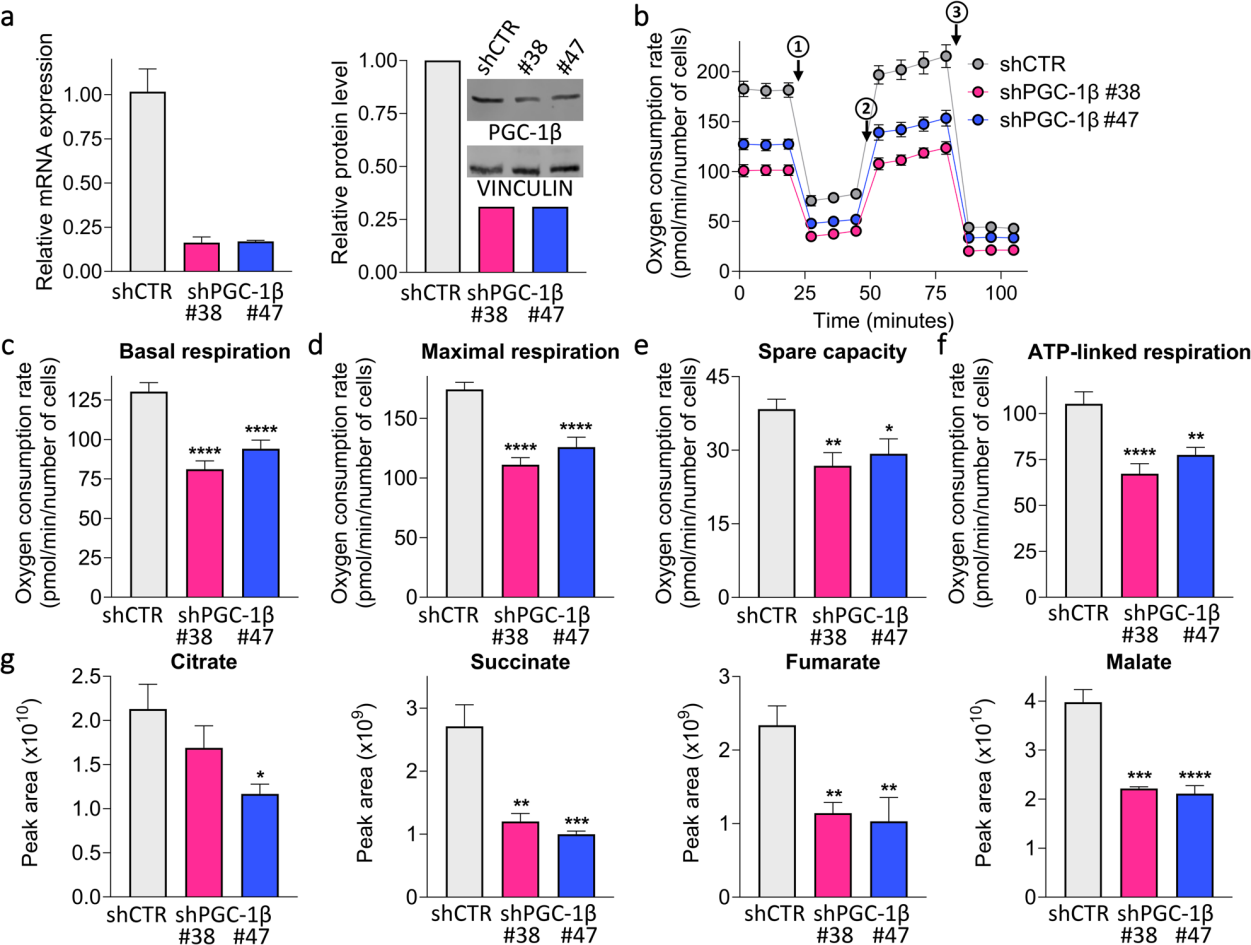
Silencing of PGC-1β impairs the oxidative metabolism of ID8 cells. **a** Relative transcript expression (left) and protein level (right) and representative western blot of PGC-1β in shPGC-1β ID8 #38 (pink) and #47 (blue) clones compared to shCTR ID8 (grey). Transcript level was normalized against β-actin and protein level against vinculin. **b**-**f** Representative Seahorse XF Cell Mito Stress Test performed in shCTR ID8, shPGC-1β ID8 #38 and #47 clones. At least 10 replicates/sample; mean ± SEM. Shown are: the oxygen consumption rate kinetic profile under basal conditions and in response to oligomycin (1), FCCP (2), Antimycin A and Rotenone (3) (**b**); basal (**c**) and maximal respiration (at 2 µM FCCP) (**d**); the spare respiration capacity (**e**) and the ATP-linked respiration (**f**). **g** Levels of intracellular intermediates of tricarboxylic acid cycle: citrate, succinate, fumarate and malate in shCTR ID8, shPGC-1β ID8 #38 and #47 clones. 3-4 replicates/sample; mean ± SEM. *p<0.05, **p<0.01, ***p<0.001, ****p<0.0001 vs. shCTR ID8. Differences were analyzed by one-way ANOVA and Dunnett’s multiple comparisons test

To investigate whether oxidative metabolism influences HR efficiency, we impaired mitochondrial activity in ID8 *Trp53*^*−/−*^-Luc cells (referred to as ID8) by silencing PGC-1β (encoded by *Ppargc1b*), a master regulator of mitochondrial biogenesis that functionally regulates mitochondrial metabolism in OC [14]. We generated isogenic PGC-1β silenced ID8 cells (shPGC-1β ID8 #38 and #47 clones) that exhibited a reduction in PGC-1β mRNA by up to 80–90% and PGC-1β protein by up to 70% compared to the control cells (shCTR ID8) (Fig. 1a). Reduction in PGC-1β levels correlated with impaired respiration capacity (measured as oxygen consumption rate (OCR); Fig. 1b), both under basal condition (Fig. 1c) and when the cells were stimulated to operate at their highest capacity (maximal respiration; Fig. 1d). PGC-1β silencing also reduced the spare respiratory capacity (Fig. 1e) and affected energetic metabolism, reducing respiration linked to ATP production compared to shCTR ID8 (Fig. 1f). Confirming the reduction in mitochondrial activity, PGC-1β silencing significantly diminished the levels of TCA cycle intermediates, particularly succinate, fumarate, and malate, without activating compensatory pathways as shown in Fig. 1g and Supplementary Fig. 1.

**Fig. 2:**
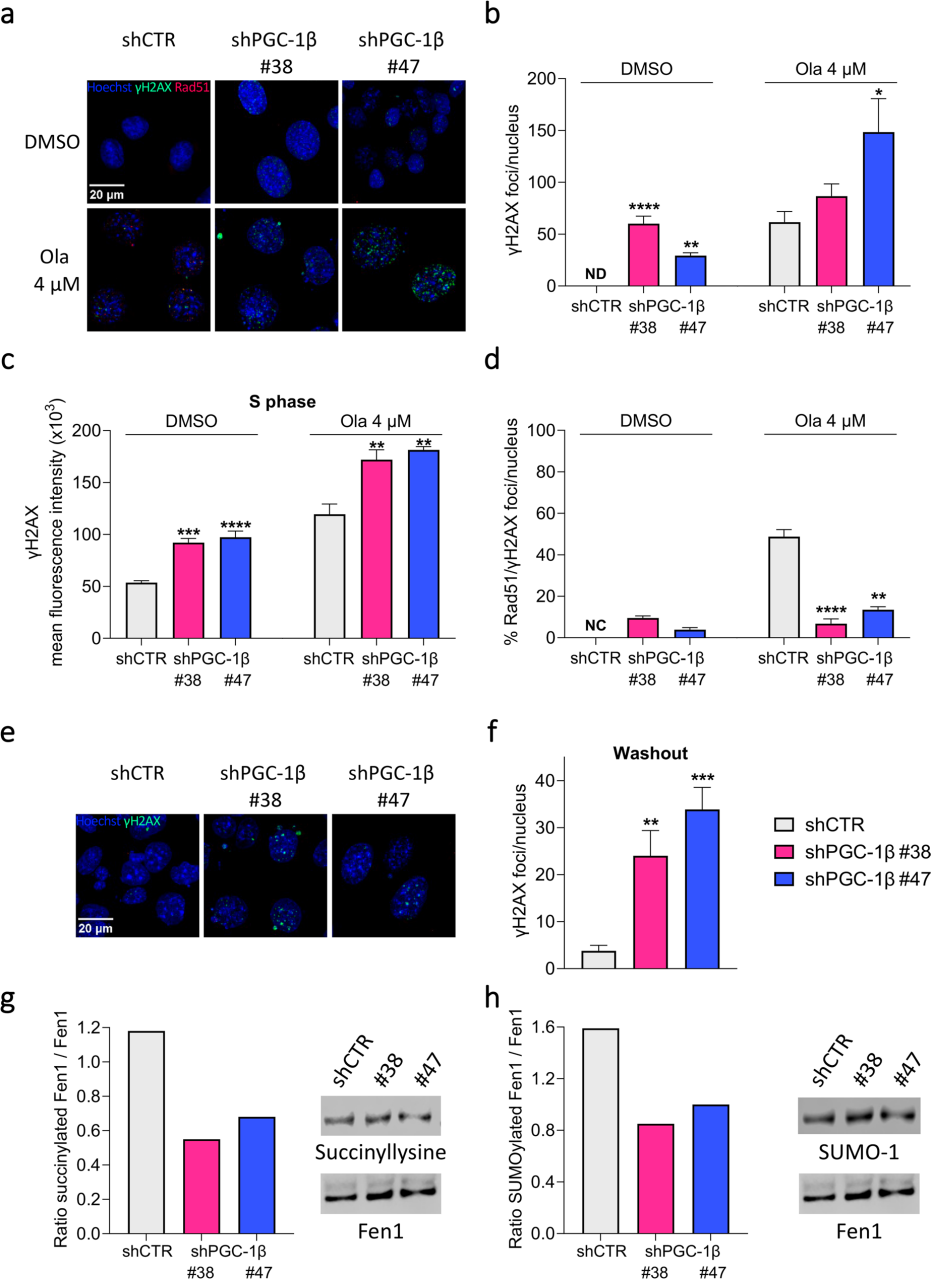
Silencing of PGC-1β impairs the repair of DNA double-strand damage by reducing Fen1 succinylation. **a**,**b** Representative images of γH2AX (green) and Rad51 (red) foci in the nuclei, stained with Hoechst (blue) (scale bar = 20 µm) (**a**) and number of γH2AX foci / nucleus (**b**) in shCTR ID8, shPGC-1β ID8 #38 and #47 clones. **c**, Mean fluorescence intensity of γH2AX in the S-phase of the cell cycle, measured by flow cytometry in shCTR ID8, shPGC-1β ID8 #38 and #47 clones treated with either DMSO or 4 µM olaparib. **d** Percentage of Rad51 foci co-localizing with γH2AX foci / nucleus in shCTR ID8, shPGC-1β ID8 #38 and #47 clones treated with either DMSO or 4 µM olaparib. At least 4 replicates/sample; mean ± SEM. *p<0.05, **p<0.01, ***p<0.001, ****p<0.0001 vs. respective treatment of shCTR ID8. **e**,**f** Representative images of γH2AX (green) foci in the nuclei, stained with Hoechst (blue) (scale bar = 20 µm) (**e**) and number of γH2AX foci / nucleus (**f**) in shCTR ID8, shPGC-1β ID8 #38 and #47 clones 24 h after removal of 4 µM olaparib treatment. 9-10 replicates/sample; mean ± SEM. **p<0.01, ***p<0.001 vs. shCTR ID8. **g**,**h** Ratio of succinylated (**g**) and SUMOylated (**h**) Fen1 in shCTR ID8, shPGC-1β ID8 #38 and #47 clones and representative western blot. Succinylated/SUMOylated Fen1 level was normalized against Fen1. Differences were analyzed by Kruskal-Wallis test and Dunn’s multiple comparisons test (**b**,**d**,**f**) and ordinary one-way ANOVA and Dunnett's multiple comparisons test (**c**). Ola = olaparib; ND = not detectable; NC = not calculable

To assess the effect of silencing on DSB formation and repair by HR, we induced DNA damage by treating shCTR and shPGC-1β ID8 cells with the PARP inhibitor olaparib. We quantified γH2AX foci to assess DNA damage and γH2AX/Rad51 co-localization to evaluate DNA repair. As expected, DNA damage accumulated in all cell lines following olaparib treatment (Ola 4 µM) compared to the control condition (DMSO treatment) (Fig. 2a-c). However, γH2AX foci were significantly augmented in shPGC-1β ID8 clones compared to shCTR ID8 cells (Fig. 2a, quantified in Fig. 2b), particularly in S-phase cells (Fig. 2c).

The increase in DSBs observed in silenced clones was associated with a reduced capacity to repair DNA damage. While approximately 50% of γH2AX foci were Rad51 positive in shCTR ID8 cells, fewer than 20% co-localized with Rad51 in shPGC-1β ID8 clones (Fig. 2a; quantified in Fig. 2d), even though total Rad51 levels were unchanged (Supplementary Fig. 2a). As a result, high levels of γH2AX foci persisted in PGC-1β silenced clones after olaparib withdrawal, whereas shCTR ID8 cells successfully repaired the damage, leaving no detectable γH2AX foci after drug removal (Fig. 2e; quantified in Fig. 2f).

Notably, spontaneous accumulation of γH2AX foci was observed in vehicle-treated shPGC-1β ID8 clones (Fig. 2a, quantified in Fig. 2b), which was associated with reduced colocalization of γH2AX and Rad51 foci (Fig. 2d). These findings indicate an intrinsic defect in DNA repair following PGC-1β silencing, even in the absence of olaparib.

To determine the underlying mechanism, we focused our attention on Fen1, a key protein involved in DNA DSB repair. Fen1 is responsible of Rad51 recruitment to DNA damage upon succinylation and consequent SUMOylation [26], thereby facilitating the repair of DSBs.

In our model system, we observed a reduction in the ratio of succinylated/total and SUMOylated/total Fen1 in shPGC-1β ID8 clones compared to shCTR ID8 cells (Fig. 2g, h and Supplementary Fig. 3), which can be correlated with reduced levels of succinate in PGC-1β silenced cells, as shown in Fig. 1g.

Taken together, these results indicate that alteration of oxidative metabolism decreases the ability of OC cells to efficiently repair DNA DSBs by reducing succinate levels and Fen1 activation, thereby preventing Rad51 recruitment.

### IACS-010759 reduces succinate and impairs DNA damage repair

**Fig. 3:**
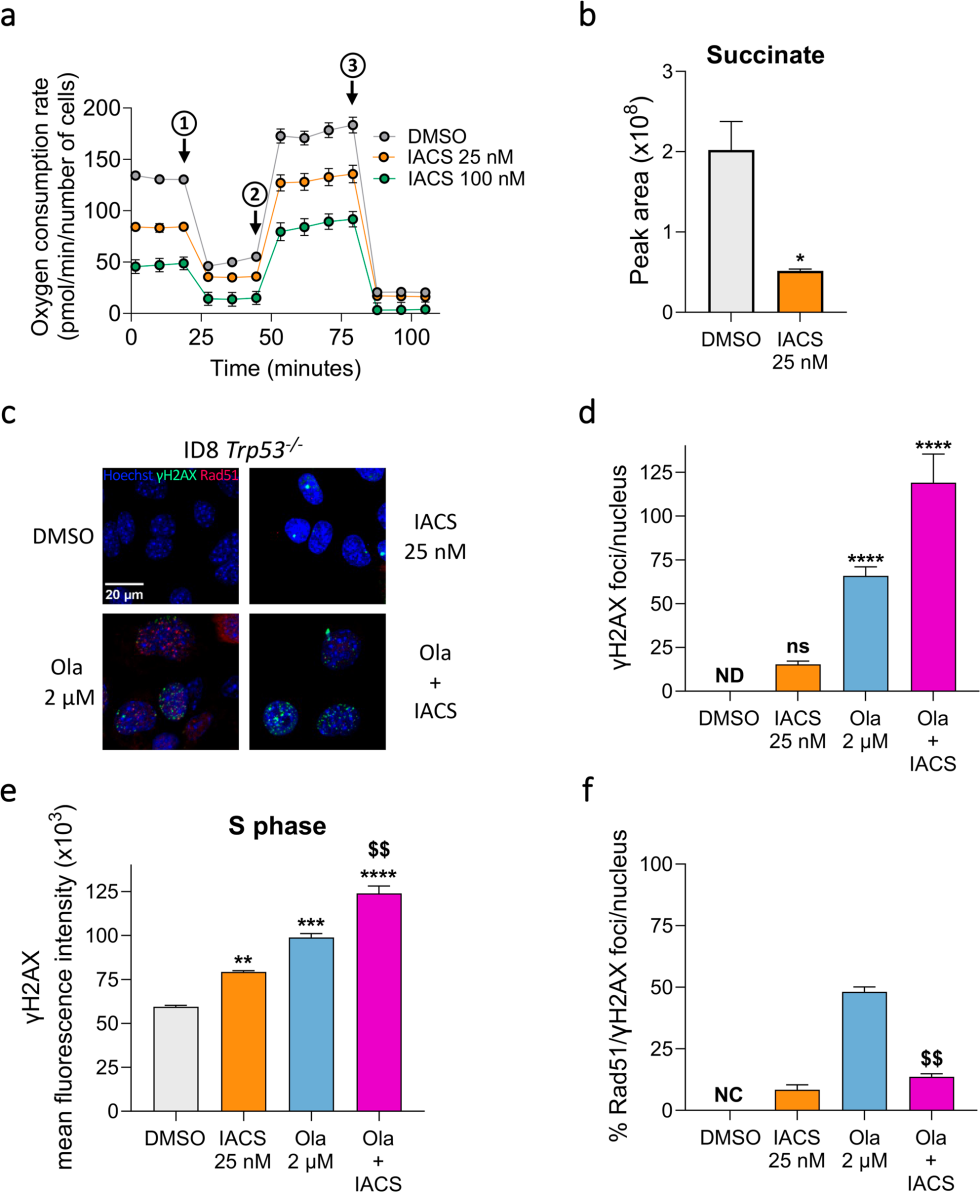
IACS-010759 impairs the oxidative metabolism and reduces the repair of DNA double-strand damage. **a** Representative Seahorse XF Cell Mito Stress Test performed in ID8 cells upon DMSO (grey), 25 nM (orange) or 100 nM (green) IACS-010759. 6 replicates/sample; mean ± SEM. The kinetic profile of oxygen consumption rate measured under basal conditions and in response to oligomycin (1), FCCP (2), Antimycin A and Rotenone (3) is shown. **b** Levels of the intracellular intermediate of tricarboxylic acid cycle succinate in ID8 cells upon DMSO or 25 nM IACS-010759. 4 replicates/sample; mean ± SEM. *p<0.05, Mann-Whitney test. **c**,**d** Representative images of γH2AX (green) and Rad51 (red) foci in the nuclei, stained with Hoechst (blue) (scale bar = 20 µm) (**c**), and number of γH2AX foci / nucleus (**d**) in ID8 upon DMSO, 25 nM IACS-010759, 2 µM olaparib (light blue) and their combination (purple). **e** Mean fluorescence intensity of γH2AX of the cells in the S-phase of the cell cycle, measured by flow cytometry in ID8 upon DMSO, 25 nM IACS-010759, 2 µM olaparib and their combination. **f** Percentage of Rad51 foci co-localizing with γH2AX foci / nucleus in ID8 upon DMSO, 25 nM IACS-010759, 2 µM olaparib and their combination. At least 4 replicates/sample; mean ± SEM. ns = not significant, **p<0.01, ***p<0.001, ****p<0.0001 vs. DMSO; ^$^^$^*p*<0.01 vs. olaparib. If not stated otherwise, differences were analyzed by Kruskal-Wallis test and Dunn’s multiple comparisons test (**d**,**f**) and ordinary one-way ANOVA and Dunnett’s multiple comparisons test (**e**). Ola = olaparib; IACS = IACS-010759; ND = not detectable; NC = not calculable

Similar results were obtained by pharmacologically targeting oxidative metabolism with the complex I inhibitor IACS-010759. IACS-010759 impaired the respiration of ID8 cells in a dose-dependent manner (Fig. 3a), reducing OCR under both basal and maximal conditions (Supplementary Fig. 4a, b), without influencing the spare capacity (Supplementary Fig. 4c). IACS-010759 also altered the energy profile, reducing the ability of cells to synthesize ATP *via* OXPHOS (Supplementary Fig. 4d). Finally, IACS-010759 significantly reduced succinate levels (Fig. 3b) and, to a lesser extent, other TCA cycle metabolites (i.e. citrate, fumarate, and malate) (Supplementary Fig. 4e).

IACS-010759 mirrored the effects of the genetic approach, inducing accumulation of γH2AX foci (Fig. 3c; quantified in Fig. 3d) - particularly in S phase cells (Fig. 3e) - and impairing Rad51 recruitment to DNA DSBs (Fig. 3c; quantified in Fig. 3f) despite unchanged Rad51 levels (Supplementary Fig. 2b). In the presence of olaparib, IACS-010759 further amplified DNA damage (Fig. 3c-e) by again reducing Rad51 loading onto DSBs. Whereas approximately 50% of olaparib induced γH2AX foci co-localized with Rad51, this proportion fell below 20% when IACS-010759 was added (Fig. 3c; quantified in Fig. 3f).

### Impairment of mitochondrial metabolism increases the sensitivity of ID8 cells to PARP inhibitors

**Fig. 4:**
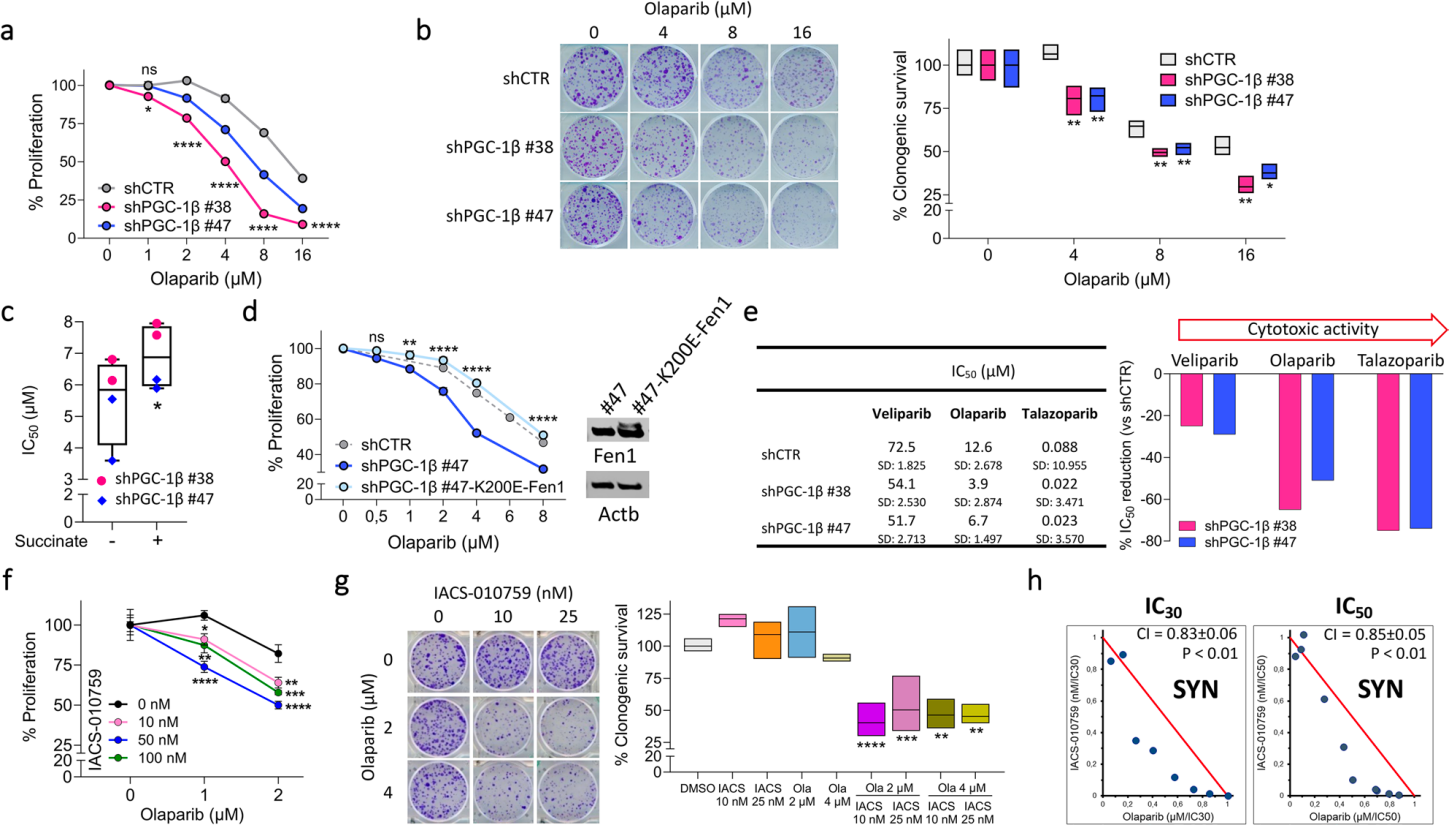
Impairment of oxidative metabolism sensitizes HRP ID8 cells to different PARP inhibitors. **a** Representative dose-response curves to olaparib of shCTR ID8, shPGC-1β ID8 #38 and #47 clones. At least 4 replicates/sample; mean ± SEM. Vehicle-treated cells = reference. *p<0.05, ****p<0.0001 vs. shCTR ID8. **b** Representative images and quantification of clonogenic assay in absence/presence of olaparib. 3-4 replicates/sample; range with line at mean. shCTR ID8 cells = reference. *p<0.05, **p<0.01 vs. shCTR ID8. **c** IC_50_ values of olaparib for shPGC-1β ID8 #38 and #47 clones in absence/presence of cell permeable 10 µM succinate calculated by fitting the dose-response curves. Data are reported as range (showing individual points) with line at median. *p<0.05; paired t test. **d** Representative dose-response curves to olaparib of shPGC-1β ID8 #47 (blue), shPGC-1β ID8 #47-K200E-Fen1 (light blue), and shCTR ID8 (dotted line, grey). Western blot of activated Fen1 overexpression in shPGC-1β ID8 #47-K200E-Fen1 is also shown. At least 4 replicates/sample; mean ± SEM. Vehicle-treated cells = reference. ns = not significant, **p<0.01, ****p<0.0001 vs. shPGC-1β ID8 #47. **e** IC_50_ values of PARP inhibitors calculated by fitting the dose-response curves (mean ± SD) and percentage of IC_50_ reduction of each PARP inhibitor for shPGC-1β ID8 clones vs. the corresponding IC_50_ value for shCTR ID8 cells taken as reference. **f** Representative dose-response curves of ID8 cells to olaparib alone and with the addiction of IACS-010759 at different concentrations. 5-6 replicates/sample; mean ± SEM. Proliferation at each corresponding IACS-010759 concentration was considered as reference. *p<0.05, **p<0.01, ***p<0.001, ****p<0.0001 vs. corresponding olaparib mono-treatment. **g** Representative images and quantification of clonogenic assay with ID8 upon DMSO, IACS-010759, olaparib and their combination. 3 replicates/sample; range with line at mean. Vehicle-treated cells = reference. **p<0.01, ***p<0.001, ****p<0.0001 vs. corresponding olaparib mono-treatment. **h** Isobologram analysis and combination index (CI) applied to ID8 cells treated with olaparib and IACS-010759. Red line separates the antagonistic (upper) from the synergistic region (lower). 6 replicates/sample. If not stated otherwise, differences were analyzed by ordinary one-way ANOVA and Dunnett's multiple comparisons test. Ola = olaparib; IACS = IACS-010759; SYN = synergism

Failure to repair DNA DSBs by impairing oxidative metabolism has been exploited to improve sensitivity to PARP inhibitors. As shown in Fig. 4a, olaparib treatment significantly reduced the proliferation of shPGC-1β ID8 clones compared to that of shCTR ID8 cells. Analogously, the ability of shPGC-1β ID8 clones to form colonies was reduced compared to shCTR ID8 cells in the presence of olaparib, although silencing of PGC-1β did not alter the proliferation and the clonogenic potential of OC cells (Fig. 4b). To demonstrate that succinate and Fen1 succinylation contribute to olaparib sensitivity, we performed rescue experiments. First, PGC-1β silenced cells were supplemented with a cell permeable form of succinate and their sensitivity to olaparib was assessed. Succinate supplementation increased the IC_50_ value of shPGC-1β ID8 clones (Fig. 4c), suggestive of reduced sensitivity to olaparib. Then, to further demonstrate that reduced Fen1 succinylation is indeed responsible of the increased sensitivity to olaparib, we transfected PGC-1β silenced cells with a plasmid encoding K200E-Fen1 [26], a mutant form of the protein that mimics the structure and function of succinylated Fen1 (as shown by western blot in Fig. 4d). This approach allowed global protein succinylation to remain suppressed while restoring “succinylation” specifically for Fen1. We observed that expression of K200E-Fen1 reduced the sensitivity of PGC-1β silenced cells to olaparib, thereby supporting our hypothesis (Fig. 4d).

Sensitization was not restricted to olaparib but could be extended to other PARP inhibitors. We tested veliparib and talazoparib, respectively endowed with the lowest and the highest cytotoxic activity among PARP inhibitors [27, 28] (Fig. 4e). Silencing PGC-1β reduced the IC_50_ values of all tested PARP inhibitors and the extent of the effect correlated with their level of cytotoxic potency (25% IC_50_ reduction for veliparib, 50% for olaparib, 75% for talazoparib) (Fig. 4e).

Accordingly, pharmacological impairment of mitochondria by IACS-010759 increased sensitivity to olaparib in ID8 cells (Fig. 4f), poorly sensitive to either PARP or OXPHOS inhibition [19]. The ability of cells to form colonies was also affected by the combination treatment. Indeed, at concentrations of either IACS-010759 or olaparib not effective as single treatment, their combination reduced the total area of colonies to approximately 50% (Fig. 4g). Isobologram analysis and the combination index indicated that IACS-010759 and olaparib produced a synergistic effect when administered in co-treatment (Fig. 4h).

In agreement with the mechanism described above, the synergistic effect of OXPHOS and PARP inhibitors was not observed in cells whose DNA DSB repair mechanism was already compromised. Indeed, ID8 *Brca2*^*−/−*^ cells [21], despite being more sensitive to both PARP and OXPHOS inhibitors than parental ID8 cells (Supplementary Fig. 5a), did not show any significant benefit from the addition of IACS-010759 (Supplementary Fig. 5b).

### The combination of OXPHOS and PARP inhibition is synergistic in HR proficient human ovarian cancer cell lines without affecting normal human cells

**Fig. 5:**
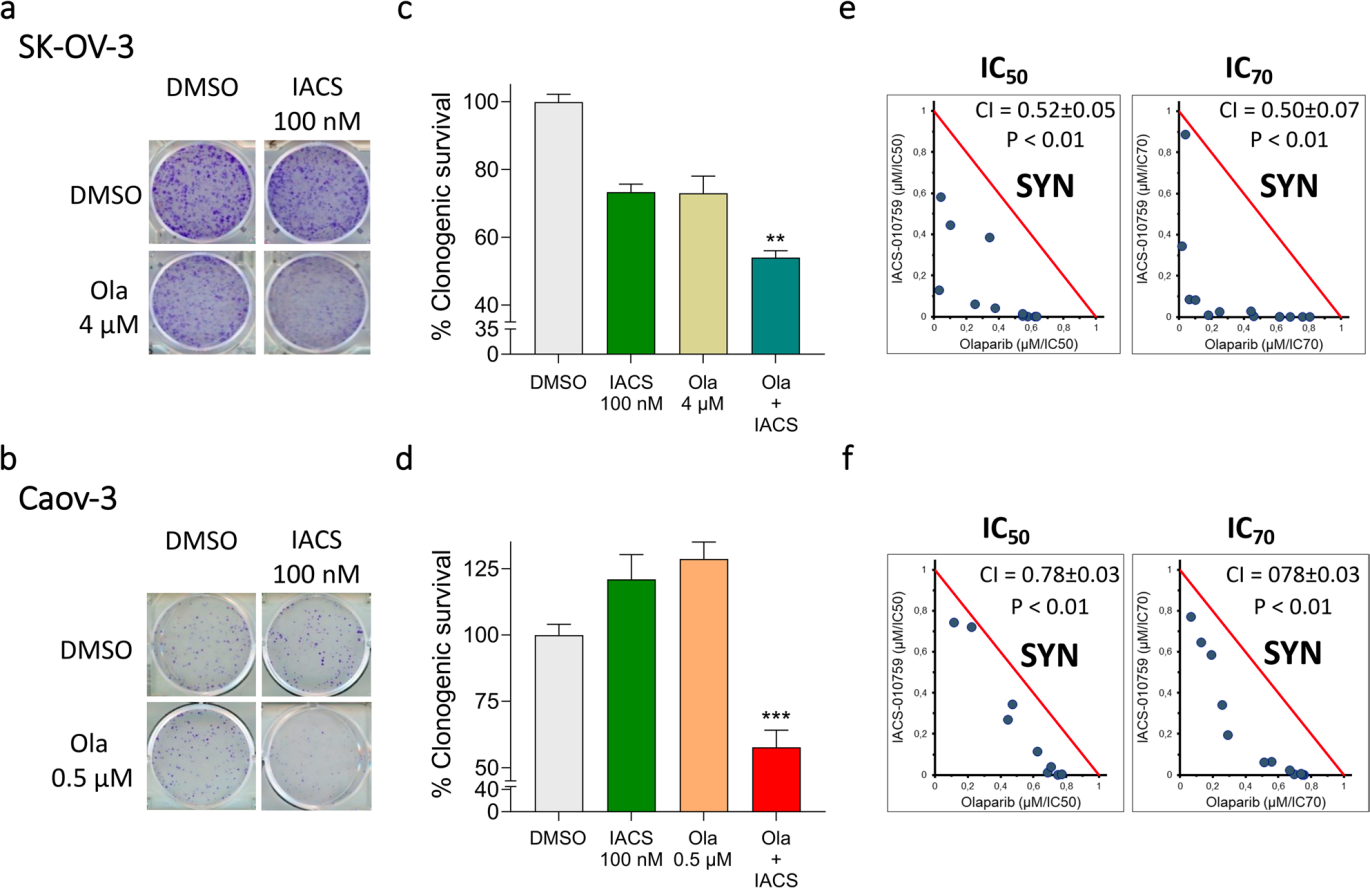
IACS-010759 increases the sensitivity of HRP SK-OV-3 and Caov-3 human ovarian cancer cells to olaparib. **a**-**d** Representative images (**a**,**b**) and quantification (**c**,**d**) of colony formation assay performed with human SK-OV-3 (**a**,**c**) and Caov-3 (**b**,**d**) cell lines upon DMSO, IACS-010759, olaparib or combined treatments at the indicated concentrations. At least 4 replicates/sample; mean ± SEM. Vehicle-treated cells = reference. **p<0.01, ***p<0.001 vs. vehicle treatment. Differences were analyzed by Kruskal-Wallis test and Dunn’s multiple comparisons test (**c**) and one-way ANOVA and Dunnett’s multiple comparisons test (**d**). **e,f** Isobologram analysis and combination index (CI) applied to SK-OV-3 (**e**) and Caov-3 (**f**) cell lines treated with olaparib and IACS-010759 in combination. Red line: additivity line that separates the antagonistic (upper) from the synergistic region (lower). 6 replicates/sample. Ola = olaparib; IACS = IACS-010759; SYN = synergism

To validate our results, we assessed the effects of IACS-010759 on the sensitivity to olaparib of HRP poorly responsive human ovarian cancer cells, i.e. SK-OV-3 and Caov-3 cell lines (olaparib IC_50_ > 15 µM). The addition of IACS-010759 to olaparib treatment impaired the ability of both cell lines to form colonies, as shown in the representative images in Fig. 5a, b. Clonogenic survival was significantly reduced upon combination treatments compared to olaparib single treatment (Fig. 5c, d). Isobologram analysis and the combination index demonstrated that olaparib and IACS-010759 produced a synergistic effect in both SK-OV-3 and Caov-3 cells when administered in combination (Fig. 5e, f).

To exclude over-toxicity in normal human cells, typically HRP, we evaluated whether the combination treatment affected the proliferation of smooth muscle cells (UASMC), fibroblasts (HESkin) and endothelial cells (HUVEC). Even though these cells displayed different sensitivities to olaparib (IC_50_ ≈ 10 µM for HUVEC, and > 50 µM for HESkin and UASMC), the addition of IACS-010759 did not significantly alter it (Supplementary Fig. 6a-c).

Altogether, these results demonstrate that pharmacological inhibition of OXPHOS is effective in increasing olaparib sensitivity in both murine and human HRP OC cells, without affecting olaparib sensitivity in normal human cells.

### Combined IACS-010759 and olaparib treatment delays ovarian cancer progression and increases the lifespan of mice bearing HR proficient OC-PDXs

Previous results pave the way for alternative approaches for using PARP inhibitors in HRP settings. To address this possibility, we tested the anti-tumor effect of the OXPHOS and PARP inhibitor combination in OC-PDX preclinical models.

HOC76 and HOC79 OC-PDXs are characterized by mutations in *TP53* and no alterations in *BRCA1* and *BRCA2* genes [29]. Once injected orthotopically (i.p.), they reproduce the clinical behavior of the patient’s disease, produce ascites, and disseminate to the organs of the peritoneal cavity [25].

**Fig. 6:**
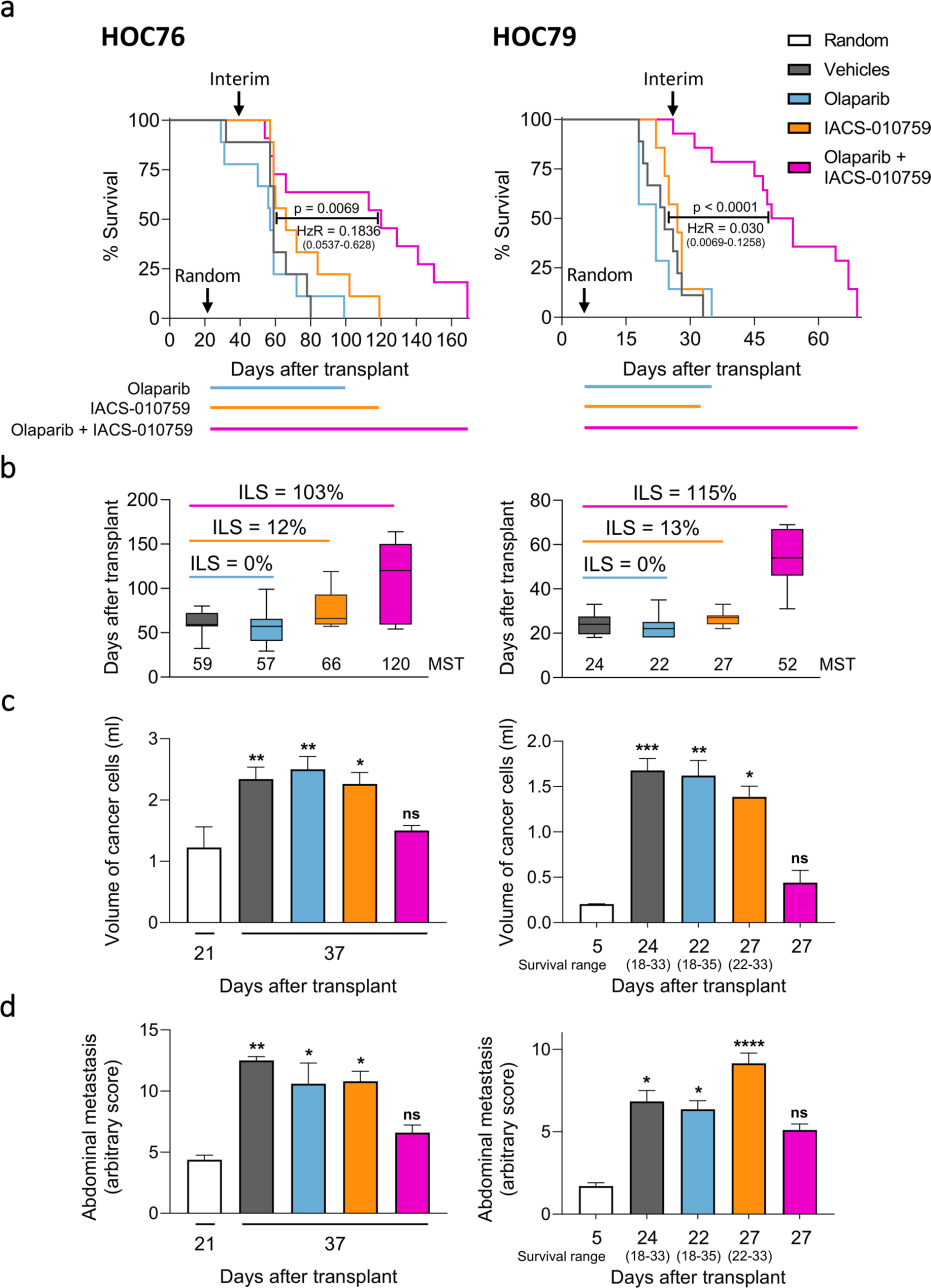
Combination of IACS-010759 with olaparib impairs tumor growth and improves survival of HRP OC-PDX-bearing mice. **a** Kaplan-Meier curves representing the percentage of OC-PDX-bearing mice survival in time, measured as days elapsed from i.p. tumor transplantation. Mice were randomized on day 21 for HOC76 (left) and day 5 for HOC79 (right) to receive either vehicles, olaparib (100 mg/kg), IACS-010759 (2.5 mg/kg), or the combined treatment in maintenance regimen until sacrifice (7-14 mice per group). Kaplan-Meier curves were compared by log-rank (Mantel-Cox) test. p-value and hazard ratio (HzR) (with confidence interval) of the combination treatment vs. vehicles are reported. **b** Median survival time (MST, days after transplant) for each treatment arm and percentage increment of lifespan (%ILS) vs. vehicle-treated group of HOC76 (left) and HOC79 (right). %ILS ≤ 40 no drug activity, 40 ≤ %ILS ≤ 100 active drug, and %ILS ≥ 100 very active drug [30]. **c**,**d** Tumor burden expressed as the volume of cell aggregates in ascites (**c**) and metastatic dissemination (**d**) evaluated at the start of treatment (i.e. day 21 for HOC76 (left) and day 5 for HOC79 (right)), and at an interim point at the third week of treatment, i.e. day 37 for HOC76-bearing mice (left) and day 27 for HOC79-bearing mice (right). Survival range is indicated for HOC79 (right). Mean ± SEM. ns = not significant, *p<0.05, **p<0.01, ***p<0.001, ****p<0.0001 vs. random. Differences were analyzed by ordinary one-way ANOVA and Dunnett's multiple comparisons test (**c**, left) and Kruskal-Wallis test and Dunn’s multiple comparisons test (**c**, right, **d**)

After randomization, OC-PDX-bearing mice were treated with vehicles, olaparib, IACS-010759, or their combination in a maintenance regimen, and survival and tumor burden were evaluated. Treatment with either IACS-010759 or olaparib resulted in no survival benefit (% increment of lifespan (%ILS) *≤* 15) (Fig. 6a, b). The combination of IACS-010759 and olaparib significantly augmented the survival of OC-PDX-bearing mice compared to the vehicle-treated group and monotherapies for both models (%ILS = 103 and 115 vs. vehicles respectively for HOC76 and HOC79) (Fig. 6a, b, for statistics refer to Supplementary Fig. 7a, b) as a consequence of tumor progression impairment (Fig. 6c, d). Indeed, interim analysis at the third week of treatment showed that malignant ascites (volume of cancer cells) was not significantly increased in mice treated with the combination compared to randomization (day 37 vs. day 21 for HOC76 and day 27 vs. day 5 for HOC79), while it was significantly higher in all other groups (Fig. 6c). Combination treatment was also effective in preventing metastasis formation compared to vehicles and monotherapies (Fig. 6d). Of note, no significant toxicity of the drug combination, evaluated as body weight, has been observed (Supplementary Fig. 8a, b).

### Combination of OXPHOS and PARP inhibitors overcomes resistance to olaparib

**Fig. 7:**
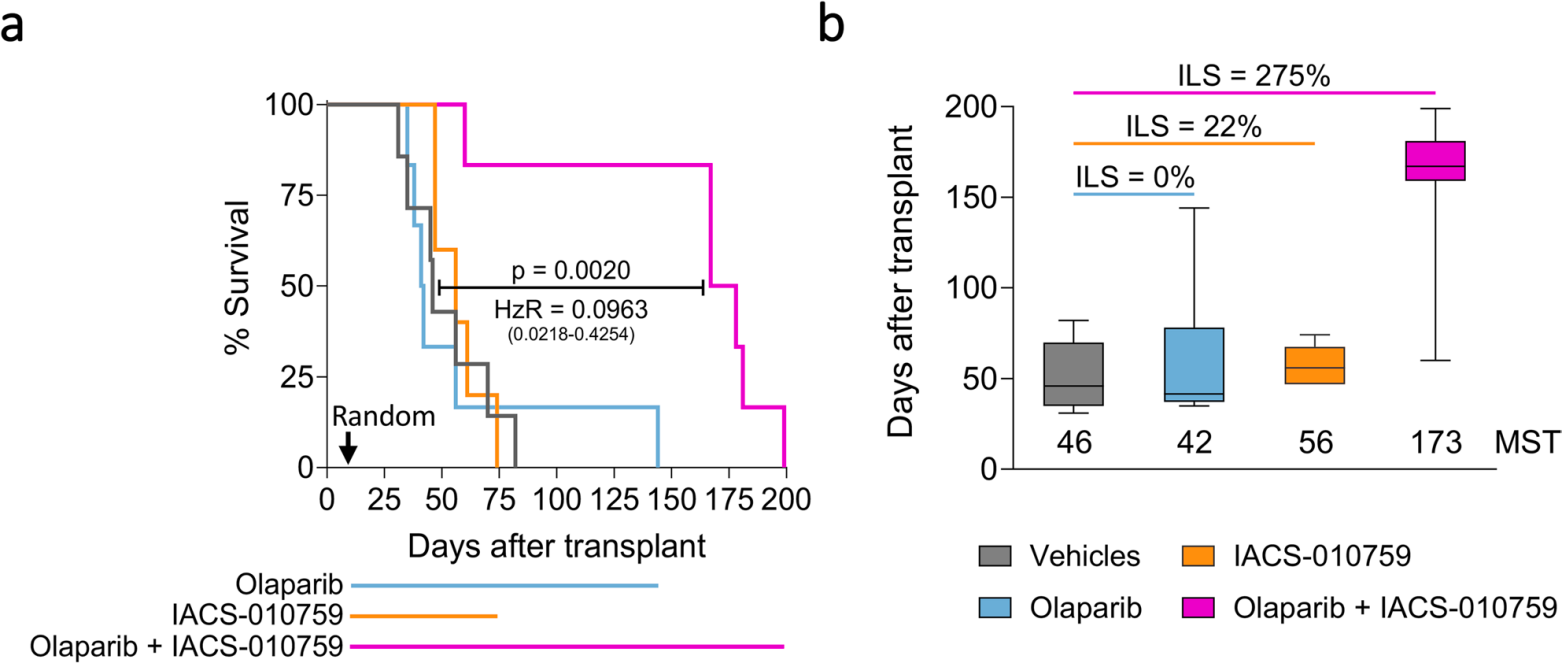
Pharmacological inhibition of OXPHOS overcomes olaparib resistance. **a** Kaplan-Meier curves representing the percentage of HOC520 OC-PDX-bearing mice survival in time, measured as days elapsed from i.p. tumor transplantation. Mice were randomized on day 10 to receive either vehicles, olaparib (100 mg/kg), IACS-010759 (2.5 mg/kg), or the combined treatment in maintenance regimen until sacrifice (5-7 mice per group). Kaplan-Meier curves were compared by log-rank (Mantel-Cox) test. p-value and hazard ratio (HzR) (with confidence interval) of the combination treatment vs. vehicles are reported. **b** Median survival time (MST) for each treatment arm and percentage increment of lifespan (%ILS) vs. vehicle-treated group of HOC520. %ILS ≤ 40 no drug activity, 40 ≤ %ILS ≤ 100 active drug, and %ILS ≥ 100 very active drug [30]

One of the major clinical challenges is how to address the unexpected resistance to PARP inhibitors in HRD ovarian tumors. We demonstrated that OXPHOS inhibition boosted PARP inhibitor efficacy also in HOC520 OC-PDX, an olaparib-resistant HRD model. Treatment with olaparib did not lead to a survival benefit compared to the vehicle group (%ILS = 0) as IACS-010759 alone (%ILS = 22) (Fig. 7a, b). Interestingly, the combination of olaparib and IACS-010759 significantly increased the lifespan of HOC520 bearing mice (%ILS = 275) (Fig. 7a, b, for statistics refer to Supplementary Fig. 7c). As in the preceding experiments, the combination treatment did not significantly affect body weight (Supplementary Fig. 8c).

Altogether, these data demonstrate that the concomitant impairment of oxidative metabolism and inhibition of PARP is an effective strategy to delay the malignant progression of HRP OC-PDXs, which are not responsive to monotherapies, and eventually to counteract intrinsic resistance to PARP inhibitors.

## Discussion

It eventually became evident that tumors can be described as a metabolic disease, where cancer cells exploit distinct metabolic and energy-generating pathways compared to the healthy cells, either as a cause or consequence of tumor progression [31]. Deregulated cancer cell metabolism may contribute to multiple traits of malignancy, such as tumor initiation, metastasis, immunosuppression, and therapy resistance [32–34], and conversely intrinsic characteristics of tumor cells can affect cell metabolism [35].

Here, we show that oxidative metabolism plays a role in the DNA damage response *via* homologous recombination (HR), and its inhibition can be exploited to improve the responsiveness to PARP inhibitors (PARPi) in HR-proficient ovarian cancers, which are currently excluded from this therapy.

By silencing PGC-1β, a key regulator of mitochondrial activity [36], we showed that impairment of oxidative metabolism affects the ability of ovarian cancer cells to efficiently repair DNA double-strand breaks. This is not due to the lack of ATP required for this energy-demanding process (Supplementary Fig. 9) but appears to be associated with the diminished availability of succinate, an intermediate of the TCA cycle. Among its diverse functions, succinate is used as a substrate to modify proteins in the cytoplasm, mitochondria, and nucleus via lysine succinylation [37], thereby modulating their activity [38]. Specifically, we found that mitochondrial impairment, by reducing succinate levels, limits the activation of Fen1, a multifunctional endonuclease essential for DNA replication and repair [26, 39]. Fen1 requires succinylation (and dependent SUMOylation by the small ubiquitin-like modifier 1 (SUMO-1)) to recruit the DNA repair machinery, including Rad51 protein [26]. Supporting our hypothesis, we showed reduced Fen1 succinylation and SUMOylation in PGC-1β silenced cells, which impaired the recruitment of Rad51 at damage foci leading to accumulation of DNA double-strand breaks. This effect is boosted in the presence of olaparib, the standard of care in the maintenance therapy of ovarian cancer [2, 3], leading to increased sensitivity of PGC-1β silenced cells to PARPi. Interestingly, testing veliparib and talazoparib, we uncover that PGC-1β silencing increased cell sensitivity to PARPi according to their level of cytotoxicity [27, 28]. This led us to speculate that the inhibition of cell proliferation was proportional to the accumulation of DNA damage, as determined by each PARPi, combined with the inability to repair them.

These results suggest the possibility of inducing an HR deficient-like phenotype through pharmacological impairment of mitochondria, highlighting a potential strategy for tumor therapy. We explored this opportunity using IACS-010759, a small molecule that inhibits the mitochondrial respiratory complex I. According to the mechanism described above, we observed that IACS-010759 induced a slight but consistent increase in DNA damage, associated with the decreased repair of spontaneously occurring DNA damage.

Furthermore, IACS-010759 prevented repair of olaparib-induced DNA damage, resulting in the synergistic effect of IACS-010759 and olaparib in ID8 *Trp53*^*−/−*^ cells. In vitro experiments confirmed that IACS-010759 addition improved the anti-proliferative effect of olaparib in a synergistic manner on a panel of HR-proficient human OC cells. Having established that the synergism between PARP and OXPHOS inhibition is associated with impaired repair of PARPi-induced DNA damage, it becomes apparent that this effect was not observed in ID8 *Trp53*^*−/−*^ *Brca2*^*−/−*^ cells, which already lack the ability to repair such damage. This is in accordance with results reported by *Lahiguera et al.* [19].

The translational relevance of our observations was further confirmed in preclinical in vivo trials employing OC patient-derived xenografts (OC-PDXs), which reproduce the tumor progression observed in patients and serve as an appropriate tool to investigate the therapeutic effect of the combination [25]. Using HR-proficient OC-PDXs, which are poorly responsive to olaparib, we observed that combined treatment with IACS-010759 and olaparib strongly delayed tumor progression and increased the survival of tumor-bearing mice compared to single agent treatment.

The combination of PARP and OXPHOS inhibition has previously been explored, primarily with biguanides such as metformin or phenformin [40–42]. These drugs, commonly used for diabetes, act on electron transport chain complex I among other targets. Supporting our results, prior studies also reported benefits from the combination strategy. However, they did not elucidate the mechanisms underlying the observed synergy. Of note, *Hijaz et al.* did not observe increased DNA damage upon metformin treatment [42], although this discrepancy may reflect the relatively weak ability of metformin to inhibit mitochondrial respiration compared with our highly specific and potent compound.

Notably, the survival benefit of IACS-010759 and olaparib combination was also observed in the HR-deficient model HOC520, mutated in *BRCA1* but not responsive to olaparib treatment [25]. This outcome deserves deeper investigation as this novel approach may allow not only the expansion of the population of patients eligible for PARPi treatment but also to address PARPi resistance in HR-deficient patients. This is a significant challenge currently faced in clinic [43]. Several agents are being studied in this context [44–46]; however, there are currently no approved therapies alone or in combination with PARPi or chemotherapy for patients whose disease progresses on PARPi [47].

In principle, the main problem with concomitant PARP and OXPHOS inhibition is the potential toxicity of this combination. Although PARPi spare HR-proficient cells [4] and we, as others [48], have observed that IACS-010759 displays no relevant activity on normal cells, toxicity has been reported in patients for both agents [49, 50]. Clinical trials employing IACS-010759 showed dose-limiting toxicities, including elevated blood lactate and neurotoxicity [49], while first-generation PARPi (including olaparib, veliparib and talazoparib) induce myelosuppression [50]. In support of this combination, we showed that normal cells were not sensitized to PARPi by OXPHOS inhibition. Moreover, no significant alteration in mice body weight and macroscopically in OXPHOS-dependent organs such as the heart, brain, and muscles were observed upon IACS-010759 in combination with olaparib. In addition, our in vitro studies indicated that the anti-proliferative effect on cancer cells was achieved by combining low doses of both agents that were not active per se, establishing a rationale for dose reduction, which could also help minimize side effects.

Relying on the molecular mechanism of the combination proposed by our findings, a possible alternative to the OXPHOS inhibitor could be the employment of small-molecule inhibitors targeting Fen1, which have recently been proposed [51]. Indeed, targeting Fen1 and its activation may allow to develop more specific and effective treatments, avoiding the adverse effects that may arise from targeting OXPHOS or other identified metabolic pathways [52, 53], which alter energy cofactors or metabolites. Supporting this possibility, *Frederick et al.* demonstrated the synergism of PARPi with Fen1 inhibitors in triple-negative breast cancer resistant to PARPi and *Berfelde et al.* uncovered how Fen1 inhibitors might enhance the DNA-damaging effect of ionizing radiation, possibly indicating radio-sensitization [54, 55].

Finally, the identification of biomarkers to select the patients, who mostly benefit from the combination treatment, would be another valuable possibility to improve the therapeutic index. Among our models, we did not observe any significant correlation of response to the combination with known cancer cell features. However it would be worthy to extend the investigation to a higher number of preclinical models to achieve the statistical power necessary to identify predictive biomarkers.

## Conclusions

In conclusion, this study identified an important piece of the complex puzzle connecting oxidative metabolism, DNA repair and responsiveness to PARPi. Here we demonstrated that mitochondrial metabolism, by affecting Fen1 succinylation, regulates DNA repair and consequently sensitivity to PARP inhibition. Our discovery is complementary to previous studies indicating that the alteration of metabolic players, including enzymes (e.g., G6PD [56]; nuclear ACLY [57]; SIRT5 [58]), metabolites (e.g., lactate and D-2-hydroxyglutarate [52, 59]) and cofactors (e.g., NADP+ [53]), influences the ability of cells to efficiently repair DNA damage by impairing (i) nucleotide production [56, 58]; (ii) the recruitment [57, 59] or functionality [53] of DNA repair proteins and (iii) the expression of genes involved in DNA repair [52]. Importantly, our results identified a specific Fen1 modification, which might be exploited as target and guide the development of treatment strategies to expand the applicability of PARP inhibitors beyond HR deficiency and aid in the management of PARPi resistance.

## Supplementary Information

Supplementary Material 1: Formenti et al_Supplementary. Supplementary figures and legends. Supplementary Figure 1. PGC-1β silencing impairs TCA cycle activity without upregulating compensatory pathways. Supplementary Figure 2. PGC-1β silencing and IACS-010759 did not alter total Rad51 amount. Supplementary Figure 3. Post-translational modifications of Fen1. Supplementary Figure 4. IACS-010759 impairs the oxidative metabolism of ID8 cells. Supplementary Figure 5. IACS-010759 addition does not alter ID8 *Brca2*^*-/-*^ cell intrinsic response to olaparib. Supplementary Figure 6. OXPHOS inhibition does not alter the sensitivity of human normal cells to olaparib. Supplementary Figure 7. Statistical analysis of in vivo experiments. Supplementary Figure 8. The treatments do not affect the body weight of OC-PDX-bearing mice. Supplementary Figure 9. PGC-1β silencing does not alter ATP level.

## Data Availability

The data generated in this study are available from the corresponding author upon request.

## Abbreviations

CI: Combination index
DSBs: Double-strand breaks
HR: Homologous recombination
HRD: Homologous recombination deficient
HRP: Homologous recombination proficient
ILS: Increment of lifespan
MST: Median survival time
OC: Ovarian cancer
OC-PDXs: Ovarian cancer patient-derived xenografts
OCR: Oxygen consumption rate
OXPHOS: Oxidative phosphorylation
PARP: Poly (ADP-ribose) polymerase
PARPi: PARP inhibitors
PGC-1β: Peroxisome proliferator-activated receptor gamma coactivator 1-beta
TCA: Tricarboxylic acid cycle
