# Supplementary material for: Impairment of oxidative metabolism compromises Rad51 recruitment and potentiates PARP inhibitor effectiveness in ovarian cancer
Source: J Exp Clin Cancer Res. 2026 Jan 14;45:45. doi: 10.1186/s13046-026-03641-6 (PMC12888472; doi:10.1186/s13046-026-03641-6)
Supplement: Supplementary file 1 — Supplementary Material 1: Formenti et al_Supplementary. Supplementary figures and legends. Supplementary Figure 1. PGC-1β silencing impairs TCA cycle activity without upregulating compensatory pathways. Supplementary Figure 2. PGC-1β silencing and IACS-010759 did not alter total Rad51 amount. Supplementary Figure 3. Post-translational modifications of Fen1. Supplementary Figure 4. IACS-010759 impairs the oxidative metabolism of ID8 cells. Supplementary Figure 5. IACS-010759 addition does not alter ID8 Brca2-/- cell intrinsic response to olaparib. Supplementary Figure 6. OXPHOS inhibition does not alter the sensitivity of human normal cells to olaparib. Supplementary Figure 7. Statistical analysis of in vivo experiments. Supplementary Figure 8. The treatments do not affect the body weight of OC-PDX-bearing mice. Supplementary Figure 9. PGC-1β silencing does not alter ATP level. [file 13046_2026_3641_MOESM1_ESM.pdf]

Supplementary Figures

Impairment of oxidative metabolism compromises Rad51 recruitment and potentiates PARP inhibitor effectiveness in ovarian cancer

Laura Formenti[1$], Francesca Abramo[1$], Giulia Dellavedova[1], Valentina Dematteis[2], Alessandra Decio[2&], Chiara Grasselli[3], Paola Fabbrizio[4], Laura Brunelli[5], Raffaella Giavazzi[2§] and Carmen Ghilardi[1§*]

Supplementary Figure 1. PGC-1β silencing impairs TCA cycle activity without upregulating compensatory pathways

Supplementary Figure 2. PGC-1β silencing and IACS-010759 did not alter total Rad51 amount

Supplementary Figure 3. Post-translational modifications of Fen1

Supplementary Figure 4. IACS-010759 impairs the oxidative metabolism of ID8 cells

Supplementary Figure 5. IACS-010759 addition does not alter ID8 *Brca2*$^{-/-}$ cell intrinsic response to olaparib

Supplementary Figure 6. OXPHOS inhibition does not alter the sensitivity of human normal cells to olaparib

Supplementary Figure 7. Statistical analysis of *in vivo* experiments

Supplementary Figure 8. The treatments do not affect the body weight of OC-PDX-bearing mice

Supplementary Figure 9. PGC-1β silencing does not alter ATP level

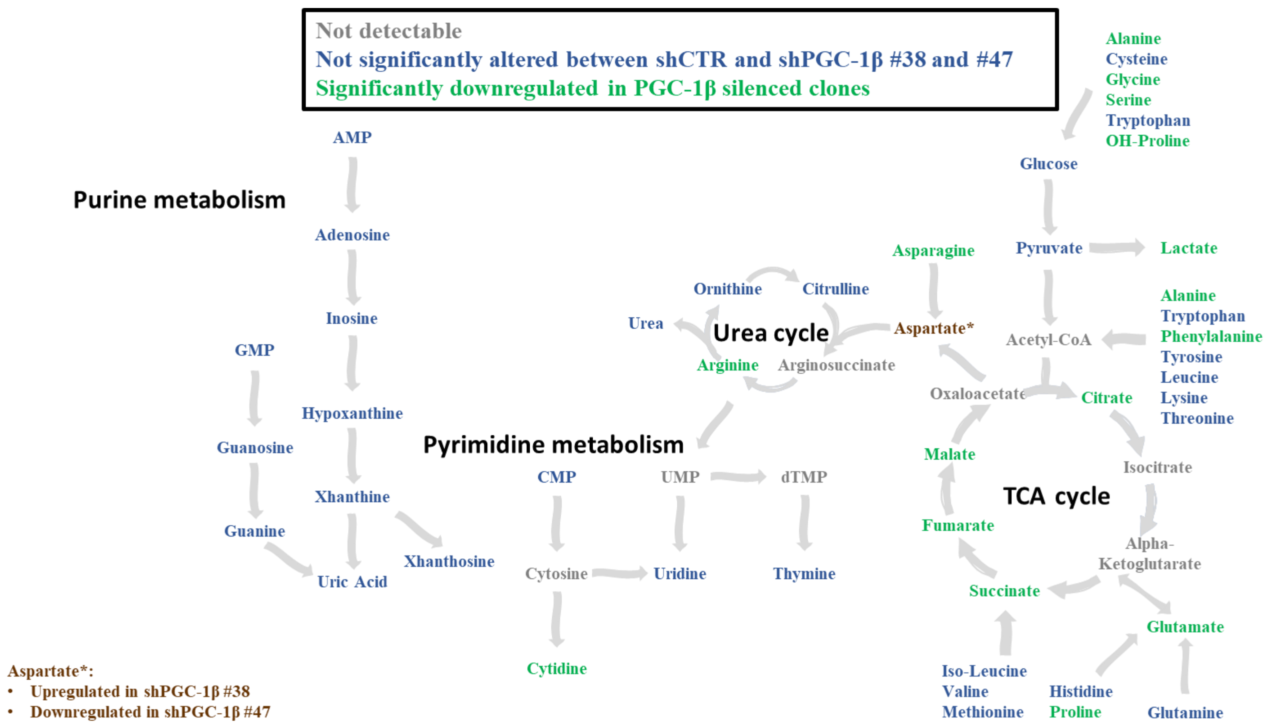

Supplementary Figure 1. PGC-1β silencing impairs TCA cycle activity without upregulating compensatory pathways

Metabolic pathway depicting the metabolites identified in shCTR ID8, shPGC-1β ID8 #38 and #47 clones (three replicates each). Green = metabolites significantly ($p<0.05$, at least) downregulated by PGC-1β silencing. Blue = metabolites not affected by PGC-1β silencing. Grey = metabolites not detectable in our analysis. Differences were analyzed by one-way ANOVA and Dunnett's multiple comparisons test.

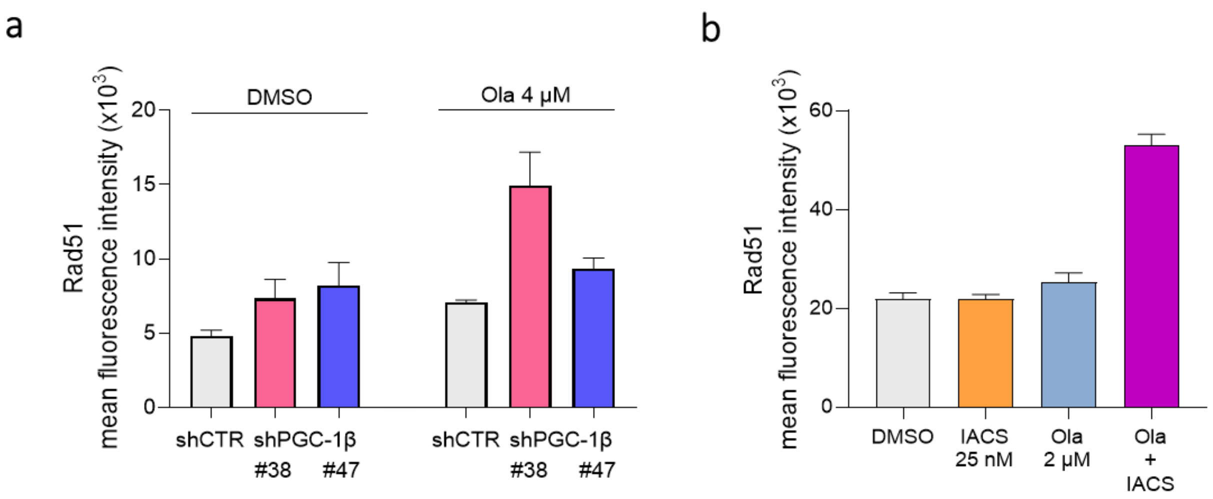

Supplementary Figure 2. PGC-1β silencing and IACS-010759 did not alter total Rad51 amount

a, Mean fluorescence intensity of Rad51, measured by flow cytometry in shCTR ID8 (grey), shPGC-1β ID8 #38 (pink) and #47 (blue) clones treated with either DMSO or 4 µM olaparib.

b, Mean fluorescence intensity of Rad51, measured by flow cytometry in ID8 upon DMSO (grey), 25 nM IACS-010759 (orange), 2 µM olaparib (light blue) and their combination (purple).

At least 4 replicates/sample; data are reported as mean ± SEM. Ola = olaparib; IACS = IACS-010759.

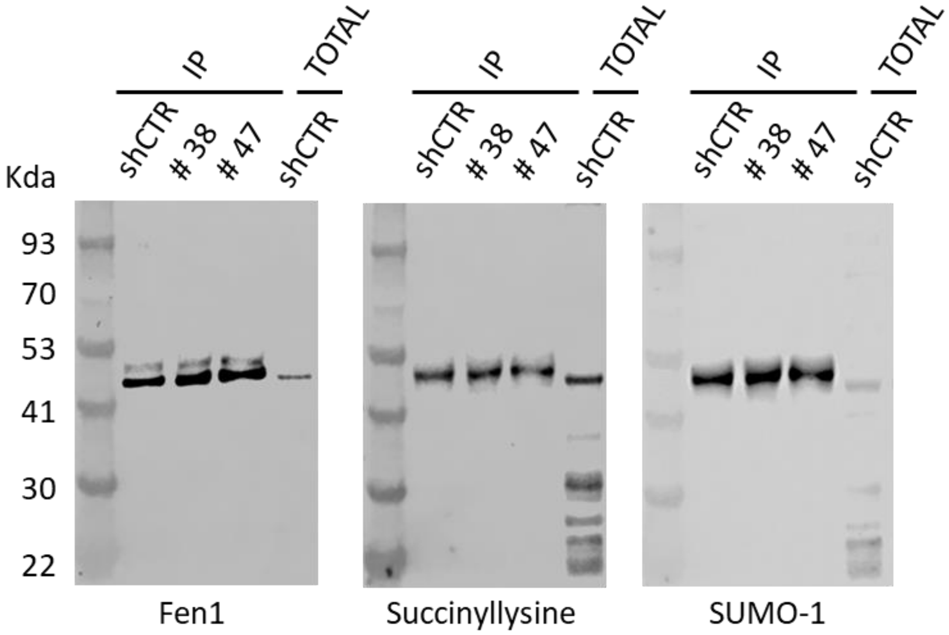

Supplementary Figure 3. Post-translational modifications of Fen1

Western Blot of immunoprecipitated Fen1 from shCTR ID8, shPGC-1β ID8 #38 and #47 clones. Total protein from shCTR ID8 were loaded as control. Fen1 immunoblot revealed two bands corresponding to unmodified Fen1 (lower band) and activated Fen1 (upper band). In the total protein lysate, only the most abundant unmodified Fen1 band can be detected. Antibodies directed against succinylated and SUMOylated proteins specifically recognize the activated form of Fen1 in the immunoprecipitated samples, while multiple bands in the total protein samples.

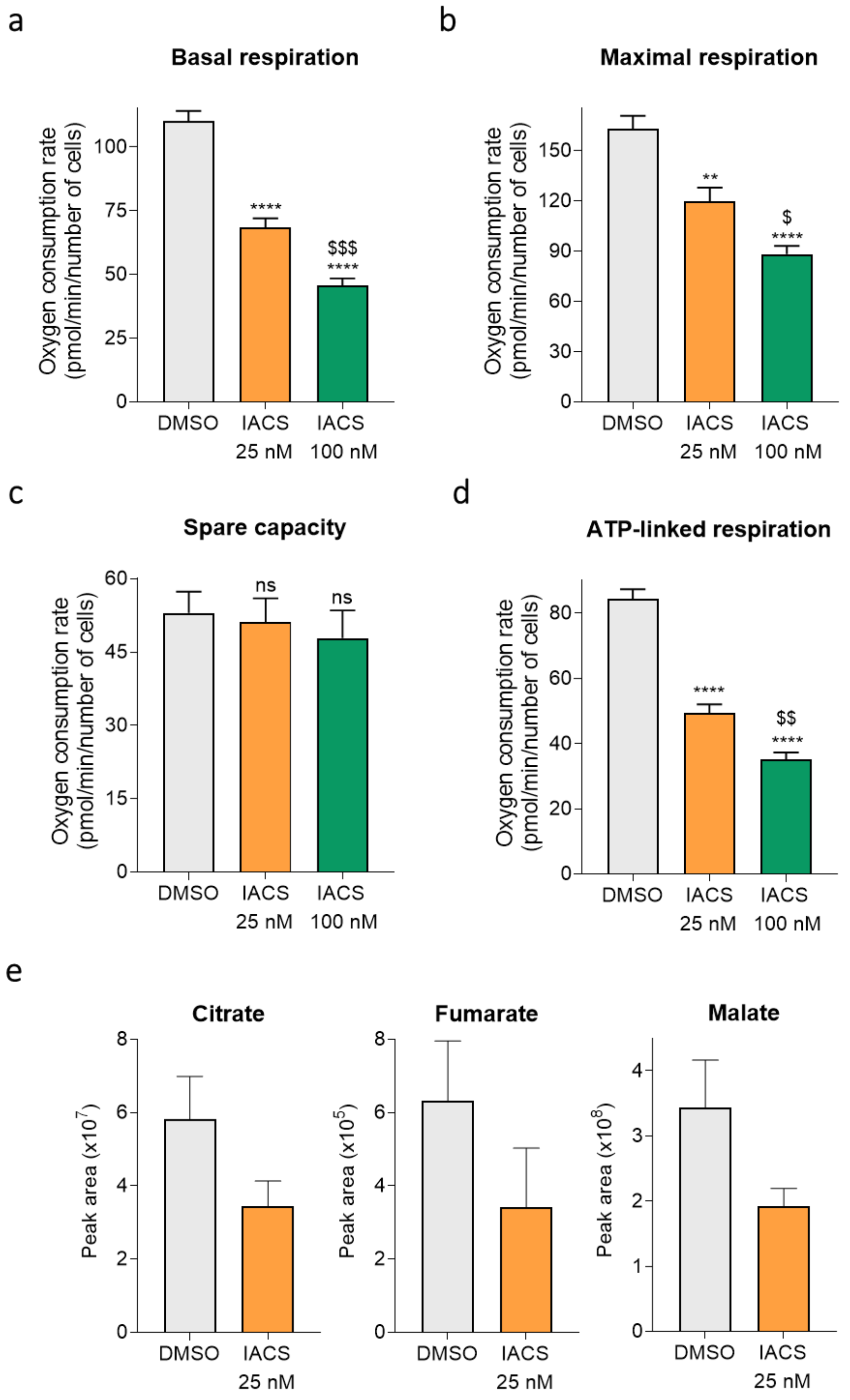

Supplementary Figure 4. IACS-010759 impairs the oxidative metabolism of ID8 cells

a-**d**, Representative Seahorse XF Cell Mito Stress Test performed in ID8 cells upon DMSO (vehicle, grey), 25 nM (orange) and 100 nM (green) IACS-010759. 6-7 replicates/sample; data are reported as mean ± SEM. Shown are: the basal (**a**) and maximal respiration (at 2 µM FCCP) (**b**); the spare respiration capacity (**c**) and the ATP-linked respiration (**d**). Differences were analyzed by ordinary one-way ANOVA and Tuckey's multiple comparisons test. ns = not significant, **p<0.01, ****p<0.0001 vs DMSO; $p<0.05, $$p<0.01, $$$p<0.001 vs 25 nM IACS-010759. IACS = IACS-010759.

e, Levels of intracellular intermediates of tricarboxylic acid cycle: citrate, fumarate and malate in ID8 cells upon DMSO (grey) or 25 nM IACS-010759 (orange). 4 replicates/sample; data are reported as mean ± SEM. IACS = IACS-010759.

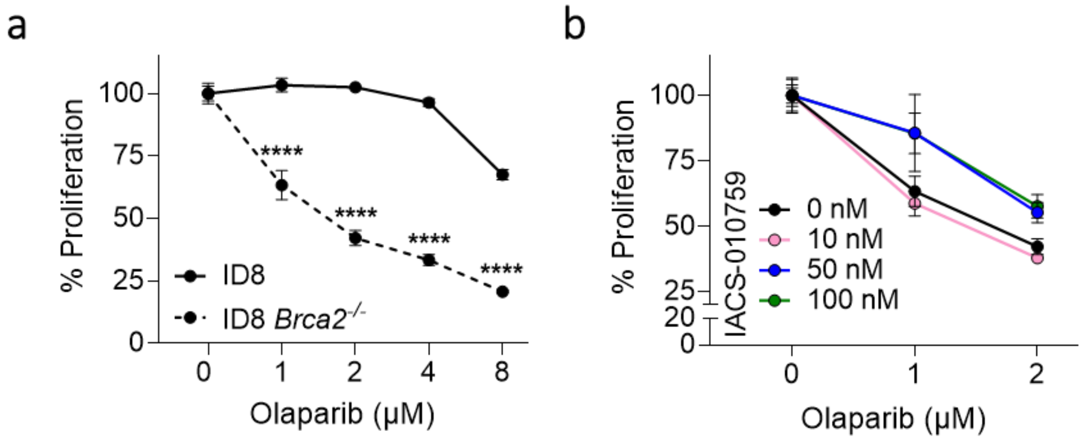

Supplementary Figure 5. IACS-010759 addition does not alter ID8 *Brca2*$^{-/-}$ cell intrinsic response to olaparib

a, Representative dose-response curves to olaparib of ID8 (solid line) and ID8 *Brca2*$^{-/-}$ (dashed line) (concentration range: 1-8 µM). 5-6 replicates/sample; data are reported as mean ± SEM. For each cell line, proliferation of vehicle-treated cells was considered as reference. Differences were analyzed by two-way ANOVA and Šídák's multiple comparisons test at each olaparib concentration. ****p<0.0001.

b, Representative dose-response curves to olaparib either alone (black) or with the addition of increasing concentration of IACS-010759 (pink: 10 nM, blue: 50 nM, green: 100 nM) of ID8 *Brca2*$^{-/-}$. 5-12 replicates/sample; data are reported as mean ± SEM. For olaparib mono-treatment, proliferation of vehicle-treated cells was considered as reference. For combination treatment, proliferation at each corresponding IACS-010759 concentration was considered as reference.

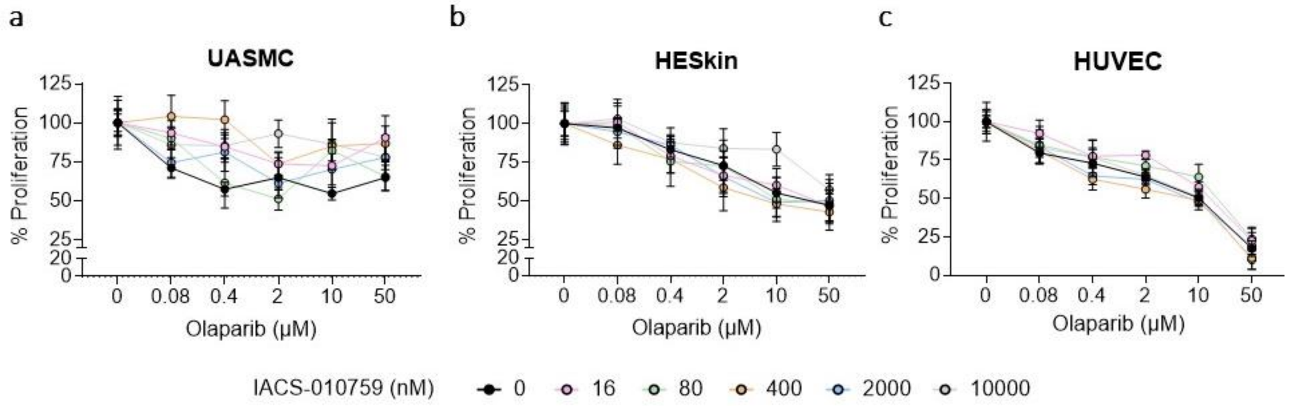

Supplementary Figure 6. OXPHOS inhibition does not alter the sensitivity of human normal cells to olaparib

a-**c**, Representative dose-response curves to olaparib either alone (black) or with the addiction of increasing concentration of IACS-010759 (concentration range: 16-10000 nM) for UASMC (**a**), HESkin (**b**) and HUVEC (**c**). 4-6 replicates/sample; data are reported as mean ± SEM. For olaparib treatment, proliferation of vehicle-treated cells was considered as reference. For combination treatment, proliferation at each corresponding IACS-010759 concentration was considered as reference.

a HOC76

**1) Vehicles vs IACS-010759**		
Log-rank (Mantel-Cox) test		
P value	0,0979	
P value summary	ns	
Are the survival curves sig different?	No	
Median survival		
Vehicle	59	
IACS-010759	66	
Ratio (and its reciprocal)	0,8939	1,119
95% CI of ratio	0,3548 to 2,252	0,4440 to 2,818
Hazard Ratio (logrank)		
Ratio (and its reciprocal)	1,941	0,5153
95% CI of ratio	0,7318 to 5,146	0,1943 to 1,366
**2) Vehicles vs olaparib**		
Log-rank (Mantel-Cox) test		
P value	0,6634	
P value summary	ns	
Are the survival curves sig different?	No	
Median survival		
Vehicle	59	
Olaparib	57	
Ratio (and its reciprocal)	1,035	0,9661
95% CI of ratio	0,4109 to 2,608	0,3835 to 2,434
Hazard Ratio (logrank)		
Ratio (and its reciprocal)	0,835	1,198
95% CI of ratio	0,3302 to 2,111	0,4736 to 3,029
**3) Vehicles vs olaparib+IACS-010759**		
Log-rank (Mantel-Cox) test		
P value	0,0069	
P value summary	**	
Are the survival curves sig different?	Yes	
Median survival		
Vehicle	59	
Olaparib+IACS-010759	120	
Ratio (and its reciprocal)	0,4917	2,034
95% CI of ratio	0,2037 to 1,186	0,8428 to 4,908
Hazard Ratio (logrank)		
Ratio (and its reciprocal)	2,666	0,3751
95% CI of ratio	0,9480 to 7,496	0,1334 to 1,055

b HOC79

**1) Vehicles vs IACS-010759**		
Log-rank (Mantel-Cox) test		
P value	0,3728	
P value summary	ns	
Are the survival curves sig different?	No	
Median survival		
Vehicle	24	
IACS-010759	27	
Ratio (and its reciprocal)	0,8889	1,125
95% CI of ratio	0,3310 to 2,387	0,4190 to 3,021
Hazard Ratio (logrank)		
Ratio (and its reciprocal)	1,469	0,6808
95% CI of ratio	0,5501 to 3,922	0,2550 to 1,818
**2) Vehicles vs olaparib**		
Log-rank (Mantel-Cox) test		
P value	0,733	
P value summary	ns	
Are the survival curves sig different?	No	
Median survival		
Vehicle	24	
Olaparib	22	
Ratio (and its reciprocal)	1,091	0,9167
95% CI of ratio	0,4063 to 2,929	0,3414 to 2,461
Hazard Ratio (logrank)		
Ratio (and its reciprocal)	0,857	1,167
95% CI of ratio	0,3152 to 2,330	0,4292 to 3,173
**3) Vehicles vs olaparib+IACS-010759**		
Log-rank (Mantel-Cox) test		
P value	<0,0001	
P value summary	****	
Are the survival curves sig different?	Yes	
Median survival		
Vehicle	24	
Olaparib+IACS-010759	51,5	
Ratio (and its reciprocal)	0,466	2,146
95% CI of ratio	0,2017 to 1,077	0,9288 to 4,958
Hazard Ratio (logrank)		
Ratio (and its reciprocal)	5,121	0,1953
95% CI of ratio	1,398 to 18,76	0,05330 to 0,7153

c HOC520

**1) Vehicles vs IACS-010759**		
Log-rank (Mantel-Cox) test		
P value	0,7476	
P value summary	ns	
Are the survival curves sig different?	No	
Median survival		
Vehicles	46	
IACS-010759	56	
Ratio (and its reciprocal)	0,8214	1,217
95% CI of ratio	0,2607 to 2,588	0,3864 to 3,836
Hazard Ratio (logrank)		
Ratio (and its reciprocal)	1,189	0,8413
95% CI of ratio	0,3819 to 3,700	0,2703 to 2,618
**2) Vehicles vs olaparib**		
Log-rank (Mantel-Cox) test		
P value	0,9838	
P value summary	ns	
Are the survival curves sig different?	No	
Median survival		
Vehicles	46	
Olaparib	41,5	
Ratio (and its reciprocal)	1,108	0,9022
95% CI of ratio	0,3725 to 3,298	0,3032 to 2,685
Hazard Ratio (logrank)		
Ratio (and its reciprocal)	1,01	0,9897
95% CI of ratio	0,3397 to 3,005	0,3328 to 2,944
**3) Vehicles vs olaparib+IACS-010759**		
Log-rank (Mantel-Cox) test		
P value	0,002	
P value summary	**	
Are the survival curves sig different?	Yes	
Median survival		
Vehicles	46	
Olaparib+IACS-010759	172,5	
Ratio (and its reciprocal)	0,2667	3,75
95% CI of ratio	0,08962 to 0,7935	1,260 to 11,16
Hazard Ratio (logrank)		
Ratio (and its reciprocal)	4,012	0,2493
95% CI of ratio	1,092 to 14,74	0,06785 to 0,9157

Supplementary Figure 7. Statistical analysis of *in vivo* experiments

a-**c**, Detailed statistical analysis of Kaplan-Meier curves of HOC76 (**a**), HOC79 (**b**) and HOC520 (**c**). Shown is the comparative analysis between vehicles and drug treatments.

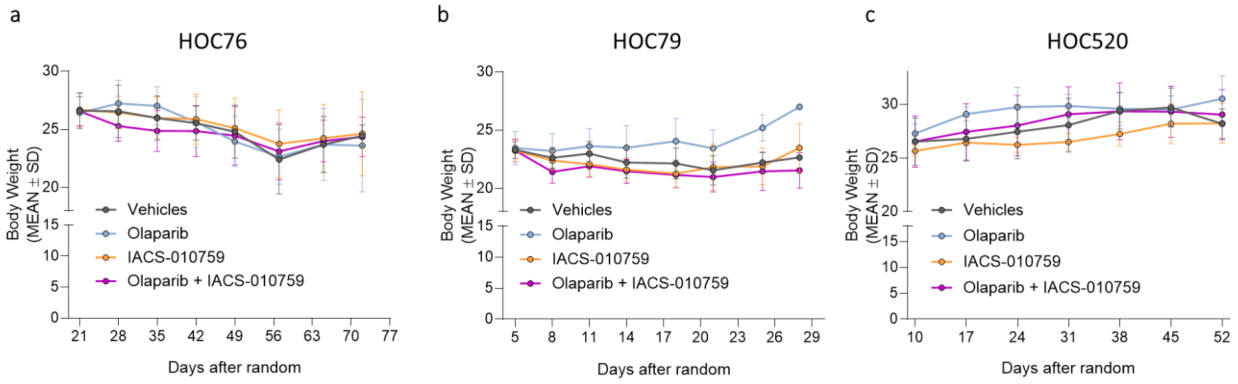

Supplementary Figure 8. The treatments do not affect the body weight of OC-PDX-bearing mice

Body weight of HOC76 (**a**), HOC79 (**b**) and HOC520 (**c**) was monitored as a rough indication of toxicity related to treatment with vehicles (grey), olaparib (light blue), IACS-010759 (orange) and their combination (purple). The graphs stop at the median survival time of vehicle and single treatment. Body weight of the mice treated with the combination remained stable until the end the experiments.

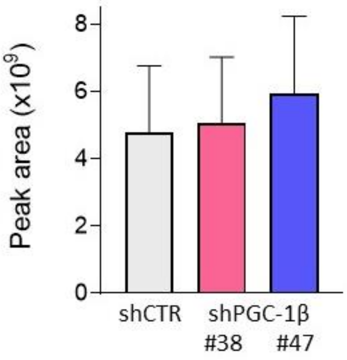

Supplementary Figure 9. PGC-1β silencing does not alter ATP level

Level of intracellular ATP in shCTR ID8 (grey), shPGC-1β ID8 #38 (pink) and #47 (blue) clones measured by mass spectrometry analysis. 12 replicates/sample; data are reported as mean ± SEM.
